# Targeted proteomics of appendicular skeletal muscle mass and handgrip strength in black South Africans: a cross-sectional study

**DOI:** 10.1038/s41598-022-13548-9

**Published:** 2022-06-09

**Authors:** Siphiwe N. Dlamini, Shane A. Norris, Amy E. Mendham, Asanda Mtintsilana, Kate A. Ward, Tommy Olsson, Julia H. Goedecke, Lisa K. Micklesfield

**Affiliations:** 1 SAMRC/Wits Developmental Pathways for Health Research Unit, School of Clinical Medicine, Faculty of Health Sciences, University of the Witwatersrand, Johannesburg, South Africa; 2 Global Health Research Institute, School of Health and Human Development, University of Southampton, Southampton, UK; 3 Health Through Physical Activity, Lifestyle and Sport Research Centre, FIMS International Collaborating Centre of Sports Medicine, Division of Physiological Sciences, Department of Human Biology, Faculty of Health Sciences, University of Cape Town, Cape Town, South Africa; 4 Non-Communicable Diseases Research Unit, South African Medical Research Council, Cape Town, South Africa; 5 Medical Research Council Lifecourse Epidemiology Centre, University of Southampton, Southampton, UK; 6 Department of Public Health and Clinical Medicine, Medicine, Umeå University, Umeå, Sweden

**Keywords:** Biomarkers, Diseases

## Abstract

Although appendicular skeletal muscle mass (ASM) and handgrip strength (HGS) are key components of sarcopenia, their underlying biological mechanisms remain poorly understood. We aimed to investigate associations of circulating biomarkers with ASM and HGS in middle-aged black South Africans. This study consisted of 934 black South Africans (469 men and 465 women, aged 41–72 years) from the Middle-aged Soweto cohort. Linear regression models were used to examine relationships between 182 biomarkers (measured with proximity extension assay) and dual-energy X-ray absorptiometry-measured ASM and dynamometer-measured HGS. Age, height, sex, smoking, alcohol, food insecurity, physical activity, visceral adipose tissue, HIV and menopausal status were included as confounders. Regression models showing sex-interactions were stratified by sex. The Benjamini–Hochberg false discovery rate (FDR) was used to control for multiple testing, and FDR-adjusted *P* values were reported. In the total sample, 10 biomarkers were associated with higher ASM and 29 with lower ASM (*P* < 0.05). Out of these 39 biomarkers, 8 were also associated with lower HGS (*P* < 0.05). MMP-7 was associated with lower HGS only (*P* = 0.011) in the total sample. Sex-interactions (*P* < 0.05) were identified for 52 biomarkers for ASM, and 6 for HGS. For men, LEP, MEPE and SCF were associated with higher ASM (*P* < 0.001, = 0.004, = 0.006, respectively), and MEPE and SCF were also associated with higher HGS (*P* = 0.001, 0.012, respectively). Also in men, 37 biomarkers were associated with lower ASM (*P* < 0.05), with none of these being associated with lower HGS. Furthermore, DLK-1 and MYOGLOBIN were associated with higher HGS only (*P* = 0.004, 0.006, respectively), while GAL-9 was associated with lower HGS only (*P* = 0.005), among men. For women, LEP, CD163, IL6, TNF-R1 and TNF-R2 were associated with higher ASM (*P* < 0.001, = 0.014, = 0.027, = 0.014, = 0.048, respectively), while IGFBP-2, CTRC and RAGE were associated with lower ASM (*P* = 0.043, 0.001, 0.014, respectively). No biomarker was associated with HGS in women. In conclusion, most biomarkers were associated with ASM and not HGS, and the associations of biomarkers with ASM and HGS displayed sex-specificity in middle-aged black South Africans. Proteomic studies should examine ASM and HGS individually. Future research should also consider sexual dimorphism in the pathophysiology of sarcopenia for development of sex-specific treatment and diagnostic methods.

## Introduction

Besides controlling locomotion, skeletal muscles play a critical role in breathing, eating, as a protein reserve, and regulating systemic metabolism, energy expenditure, and homeostasis^1^. Consequently, low skeletal muscle mass and function/quality are associated with adverse health outcomes^2^. Age-related skeletal muscle loss is common in the older persons, and with the life expectancy of humans increasing globally, diseases such as sarcopenia are becoming more prevalent^3,4^.

Several criteria for the diagnosis of sarcopenia and its components have been proposed, and all require measurement of both appendicular skeletal muscle mass (ASM) and muscle strength, which is a proxy measure of skeletal muscle function^5^. Handgrip strength (HGS) is a reliable measure of isometric muscle force, strongly correlates with ASM and is also a predictor of future mortality^6–8^. Identifying novel biomarkers associated with ASM and HGS has the potential to improve the biological understanding of sarcopenia and lead to novel intervention targets.

At a physiological level, several signaling pathways regulate skeletal muscle mass and function^9^. Accordingly, circulating biomarkers in these pathways have been associated with components of sarcopenia in cohorts of predominantly European ancestry. These include biomarkers involved in growth, the inflammatory response, neuromuscular junction, endocrine system, muscle protein turnover, and behavior-mediated pathways^10–12^. However, the majority of studies that investigated protein biomarkers of sarcopenia and/or its components have focused on relatively few candidate proteins^10–12^. Due to the complexity of the disease, exploring a wider range of potential biomarkers may be important for identifying novel biomarkers of low ASM and/or HGS^13–15^.

Most of the proteomic studies of sarcopenia and its components used discovery mass-spectrometry and were limited to relatively invasive muscle biopsies methods, with small sample numbers (18 to 58 participants)^16–18^. A recent study from Germany used proximity extension assay-measured proteomics to investigate circulating biomarkers of low ASM and high fat mass in 756 men and 722 women of European ancestry, and although they identified novel circulating biomarkers associated with ASM, they did not explore sex-specific associations^19^. While sex-interactions in complex diseases are often not explored, accumulating evidence suggests that they play a key role in determining disease risk in humans^20^. The primary aim of this study was to investigate associations of circulating biomarkers with ASM and HGS in middle-aged black South Africans. We also investigated whether sex-specificity existed in these relationships.

## Methods

### Study population and sampling

All participants were from the Middle-aged Soweto cohort (MASC), a longitudinal cohort of black middle-aged men and women from Soweto, an urban township in Johannesburg, South Africa. The MASC participants were initially recruited between 2011 and 2015 as part of the African WITS-IN-DEPTH Partnerships for Genomic Research study, which is described elsewhere^21^. The follow-up assessment was completed between January 2017 and August 2018 and samples collected were analyzed by targeted proteomic analyses, forming the basis of this cross-sectional analysis. For this study, 1021 participants (502 men and 519 women) were recruited.

Data included socio-demographic and medical questionnaire data, anthropometry and dual-energy x-ray absorptiometry-derived body composition, physical activity, HGS, and targeted plasma proteomics. Participants who reported having any form of illness within the 7 days prior to testing (n = 81) were excluded. Six participants who did not have sufficient plasma for proteomic analysis were also excluded. The final study sample comprised 469 men and 465 women.

### Questionnaire data and HIV testing

Socio-demographic and medical data were collected via an interviewer-administered questionnaire using a computer-assisted personal interviewing mode. Participants were asked about their age, which was then confirmed using the date of birth from national identity cards when available. Participants who reported currently smoking any tobacco products, including cigarettes, cigars, or pipes, were classified as smokers. Participants who reported being current consumers of any alcohol-containing drinks were classified as alcohol drinkers. HIV status was determined via an HIV antibody screen test (Guangzhou Wondfo Biotech, Guangzhou, China). To determine the menopausal status of the women, the dates of the last menstrual period were recorded, and the participants were categorized into pre-, peri-, early post-, or late post-menopausal stages^22^. Women who had undergone a hysterectomy or who were on contraceptives or hormone replacement therapy were not classified.

Food insecurity was assessed using the Household Food Insecurity Access Scale (HFIAS), a continuous estimate of food access in the participant’s household over the previous 30 days^23^. HFIAS created scores from responses to nine validated questions, which were then summed to create a total food security score with a maximum value of 27. The higher the score, the more food insecurity the household experienced in the previous 30-day period^23^.

### Anthropometry and body composition

With minimal clothing and no shoes, height was measured using a wall-mounted stadiometer (Holtain, Wales, UK), and weight was measured using a calibrated TBF-410 digital scale (Tanita Corporation, Illinois, USA). From these measurements, body mass index (BMI) was calculated as weight (kg)/height (m^2^). Waist circumference was measured using a stretch-resistant measuring tape (SECA, Hamburg, Germany) at the midpoint between the lowest rib and the iliac crest.

The QDR 4500A dual-energy X-ray absorptiometry (DXA) (Hologic, Bedford, USA) was used to measure whole-body composition, and the data were analyzed with APEX software version 13.4.2.3. The arm replacement method was used for individuals who could not fit within the scan border^24^. DXA data included the subtotal (total body minus head) body fat mass and visceral adipose tissue (VAT), and fat-free soft tissue mass of the arms and legs was summed to determine ASM.

### Physical activity and HGS

Total physical activity was measured using the integrated signals from two accelerometers, which quantified total movement volume (Euclidian norm minus one (ENMO), expressed in milli-g (m*g*)). The GTX3 + ActiGraph (ActiGraph LLC, Pensacola, USA) was fitted on the right hip, while the ActivPAL (PAL Technologies Ltd., Glasgow, Scotland) was fitted on the mid anterior right thigh of the participants. The participants were asked to wear both accelerometers simultaneously for seven consecutive 24-h days and to record their sleeping (including napping) times. To assess HGS, three grip strength measurements from the non-dominant arm were obtained while seated, with the arm flexed at 90° next to the body, using a Jamar hydraulic handheld dynamometer (Sammons Preston, Bolingbrook, USA). The maximum value was recorded and used in this study.

### Definition of sarcopenia and its components

The Foundation for the National Institutes of Health guidelines were used to define low ASM, low HGS, and sarcopenia^5^. To justify the use of BMI-adjusted cut-points for the definition of sarcopenia and its components, both BMI-adjusted and unadjusted definitions of low ASM and HGS were considered. The BMI-unadjusted cut-points for low ASM were < 19.75 kg in men and < 15.02 kg in women, and for low HGS were < 26 kg in men and < 16 kg in women. In contrast, the BMI-adjusted cut-points for low ASM were < 0.789 in men and < 0.512 in women, and for low HGS were < 1.0 in men and < 0.56 in women. The definition of sarcopenia was determined as the presence of both BMI-adjusted low ASM and BMI-adjusted low HGS.

### Blood sampling and proteomics

Plasma was obtained from 10–12 h overnight fasting blood samples and targeted proteomic analyses were performed using OLINK proteomics AB (Uppsala, Sweden). OLINK proteomic analyses use proximity extension assay technology, and the details of the method have been described^25^. In this study, the plasma samples were measured on cardiovascular disease (CVD) panels II and III, which included 184 biomarkers (www.olink.com/downloads) that are related to the following pre-classified biological processes: angiogenesis, blood vessel morphogenesis, catabolic process, cell adhesion, coagulation, heart development, immune response, inflammatory response, mitogen-activated protein kinase (MAPK) cascade, platelet activation, proteolysis, regulation of blood pressure, response to hypoxia, response to peptide hormone, wound healing, and other gene ontology (GO) terms. Notably, most biomarkers are related to multiple biological processes. The proteomic data are reported as normalized protein expression (NPX) values, which are arbitrary units on a log2 scale. To monitor the performance of the proteomic assay, four internal standards were added to each plasma sample, and the samples were randomly placed in 96-well plates. For each protein, the limit of detection was based on the mean value of negative controls plus three standards deviations calculated from the OLINK large datasets. Two protein biomarkers (BNP and SPON1) were below the detection limit and were excluded from this study.

### Statistical analysis

Statistical analyses were conducted using R software (Version 4.1.1). Missingness was calculated as (non-missing observations/total number of samples) × 100% (Additional Fig. 1). Data exploration revealed no evidence of non-random missingness. Normality of the continuous variables was assessed using histograms and box plots and the Shapiro–Wilk test. None of the continuous data were normally distributed. Therefore, all are presented as median (25th–75th percentiles), and sex differences were tested using the Wilcoxon rank-sum test. The categorical data were presented as observations (n) /total non-missing observations (N) and percent: (n/N (%)), and a chi-square test was used to assess statistical differences between sexes.

Prior to inclusion in the regression models, the Log10 mathematical transformation was used to normalize ASM, and the ordered quantile was used for HGS. Linear regression models were used to examine the associations between the biomarkers and outcomes (ASM and HGS), while adjusting for age, height, sex, smoker (yes or no), alcohol consumer (yes or no), food insecurity score, total physical activity (ENMO), VAT, and HIV status. Sex interactions were tested in all linear regression models, and only models showing significant sex interactions (*P* value for sex interaction < 0.05) were stratified and compared by sex. Menopausal status was included as an additional confounder in linear regression models that included women only. The Benjamini–Hochberg false discovery rate (FDR) was used to control for multiple testing in all linear regression models^26^. The presence of linear relationships between biomarkers and outcomes were confirmed using scatter plots. The absence of multicollinearity within each model was confirmed by assessing the variance inflation factor values (all < 3.0). The validity of each linear regression model was then confirmed by assessing the normality of the residuals.

Finally, the protein biomarkers that were associated with ASM or HGS in the linear regression models were selected, and their expression levels were compared between normal and low ASM groups or between normal and low HGS groups using the Wilcoxon rank-sum test.

### Ethics approval and consent to participate

This study was conducted according to the guidelines laid down in the Declaration of Helsinki and all procedures involving research study participants were approved by the University of the Witwatersrand Human Research Ethics Committee (Medical) (Ethics clearance M160604). After a full explanation of the purpose and nature of all procedures used, written informed consent was obtained from all participants.

## Results

Characteristics of the study sample are presented and compared by sex in Table 1. Men were taller with greater ASM and HGS than women. However, women presented with higher weight, BMI, waist circumference, body fat mass percentage, and VAT than men. The prevalence of HIV infection was 19.5% in the total sample and did not differ by sex. Likewise, the prevalence of low HGS did not differ by sex, but was 5.3% when using the BMI-adjusted, and 0.8% when using the unadjusted cut-points in the total sample. The prevalence of men with low ASM was higher than women when using the BMI-unadjusted cut-points (16.0% vs 9.3%), but not when using the BMI-adjusted cut-points. The prevalence of sarcopenia for the total samples was 1.5% and did not differ by sex. Men were more physically active, and more men smoked (48.7% vs 6.5%) and consumed alcohol (73.1% vs 30.0%) than women, and the HFIAS total score was significantly higher in men than women (Table 1).

**Table 1:** Characteristics of the study sample.

	All (n = 934)	Men (n = 469)	Women (n = 465)	*P*
**Age** (years)	54 (49–59)	53 (48–59)	54.0 (50–59)	0.084
**Body composition**				
Height (cm)	164.5 (157.9–171.7)	171.4 (166.9–175.3)	158.0 (154.0–162.0)	** < 0.001**
Weight (kg)	78.1 (65.5–91.2)	73.2 (60.8–86.1)	81.2 (71.7–95.7)	** < 0.001**
BMI (kg/m^2^)	29.1 (23.7–34.5)	25.3 (21.0–29.6)	33.1 (28.9–37.8)	** < 0.001**
Waist circumference (cm)	95.0 (84.8–104.5)	93.3 (82.5–104.0)	93.4 (87.8–104.5)	**0.006**
Fat mass (%)	36.0 (26.1–45.3)	26.3 (20.8–30.8)	45.3 (41.8–48.5)	** < 0.001**
Visceral adipose tissue, VAT (cm^2^)	92.0 (58.8–126.3)	78.0 (50.0–117.3)	105.6 (70.5–134.1)	** < 0.001**
ASM (kg)	21.7 (18.9–25.1)	23.8 (20.8–26.9)	19.9 (17.4–22.5)	** < 0.001**
**Prevalence of low ASM (n/N (%))**				
BMI Unadjusted (< 19.75 kg for men, < 15.02 kg for women)	121/892 (18.4)	80/449 (16.0)	41/443 (9.3)	** < 0.001**
BMI Adjusted (< 0.789 for men, < 0.512 for women)	69/892 (7.7)	30/449 (6.7)	39/443 (8.8)	0.289
**Handgrip strength, HGS (kg)**	36.0 (28.0–46.0)	45.0 (40.0–50.0)	29.0 (24.0–32.0)	** < 0.001**
**Prevalence of low HGS (n/N (%))**				
BMI Unadjusted (< 26 kg for men, < 16 kg for women)	7/916 (0.8)	6/466 (1.3)	1/450 (0.2)	0.141
BMI Adjusted (< 1.0 for men, < 0.56 for women)	46/916 (5.3)	19/466 (4.1)	27/450 (6.0)	0.238
**Prevalence of sarcopenia (n/N (%))**	13/874 (1.5)	8/447 (1.8)	5/428 (1.2)	0.628
**Prevalence of HIV infection (n/N (%))**	182/934 (19.5)	92/469 (19.6)	90/465 (19.4)	0.986
**Menopausal stage (n (%))**				
Pre-menopause			79 (17.1)	
Perimenopause			62 (13.4)	
Early post-menopause			252 (54.6)	
Late post-menopause			69 (14.9)	
**Lifestyle factors**				
Current smokers (n/N (%))	258/932 (27.7)	228/468 (48.7)	30/464 (6.5)	** < 0.001**
Current alcohol consumers (n/N (%))	481/932 (51.6)	342/468 (73.1)	139/464 (30.0)	** < 0.001**
Total physical activity (milli-*g*)	12.8 (10.4–15.8)	14.1 (11.0–17.4)	11.9 (9.9–14.1)	** < 0.001**
**HFIAS total score**	11 (9–17)	11 (9–18)	10 (9–16)	**0.018**

Continuous variables are presented as median (Interquartile Range), and categorical data are presented as observations/total non-missing observations (n/N (%)). A Wilcoxon rank-sum test was used to compare the continuous variables statistically, while a chi-square test was used to compare the categorical variables. *P* values < 0.05 are indicated in bold. n: Number of observations; P: *P* value for the statistical test; BMI: Body Mass Index; HIV: Human Immunodeficiency Virus; HFIAS: Household Food Insecurity Access Scale; ASM: Appendicular skeletal muscle mass. Missingness for all reported variables is shown in Additional Fig. 1. Menopausal stage was based on date of last menstrual period. Postmenopausal women were further classified into early postmenopausal group if they had not had a drop of blood for more than a year but less than 6 years, and late postmenopausal group if they had not had a drop of blood for more than 6 years^22^.

### Associations with ASM

#### Total sample

**Figure 1:**
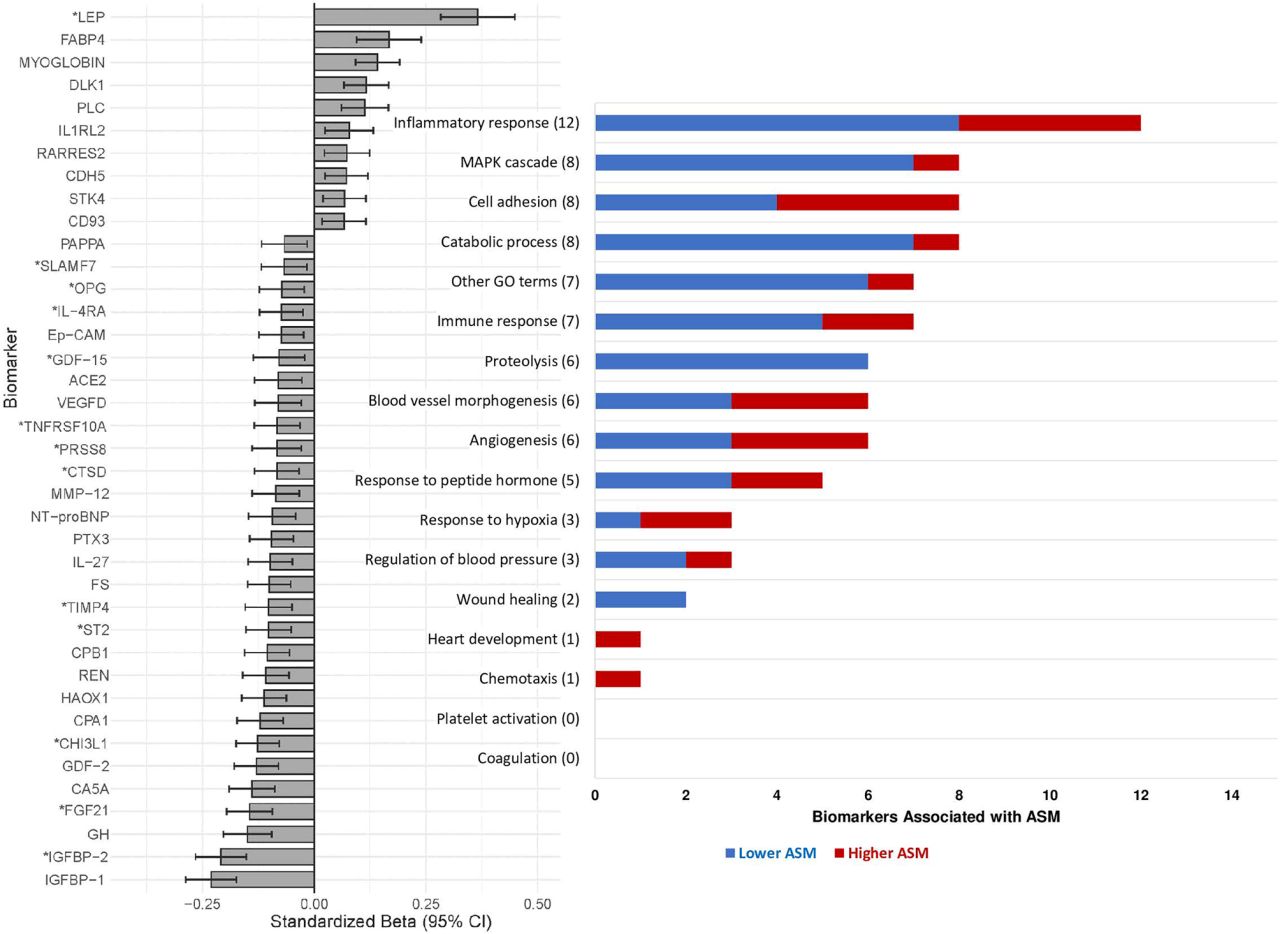
Associations between biomarkers and ASM in the total sample. Only the 39 significant biomarkers are shown. Linear regression models were adjusted for age, height, sex, smoking, alcohol, HFIAS total score, total physical activity (ENMO), visceral adipose tissue, and HIV status. *Significant sex-interaction; Standardized Beta: Standardized Beta coefficient; 95% CI: 95% Confidence Intervals. Full names of all biomarkers are listed in Additional Table 1. The numbers in brackets represent total biomarkers associated with the outcome and related to the respective biological process. The list of biomarkers associated with the biological processes are based on the OLINK website (www.olink.com/products-services/target). Chemotaxis (1): RARRES2. Heart development (1): STK4. Wound healing (2): MMP-12, IGFBP-1. Regulation of blood pressure (3): ACE2, REN, LEP. Response to hypoxia (3): VEGFD, LEP, MYOGLOBIN. Response to peptide hormone (5): TIMP4, GH, IGFBP-1, LEP, RARRES2. Angiogenesis (6): VEGFD, CHI3L1, GDF-2, LEP, PLC, STK4. Blood vessel morphogenesis (6): VEGFD, CHI3L1, GDF-2, LEP, PLC, STK4. Proteolysis (6): ACE2, TNFRSF10A, MMP-12, TIMP4, REN, CPA1. Immune response (7): SLAMF7, IL-4RA, TNFRSF10A, PTX3, IL-27, LEP, IL1RL2. Other GO (Gene Ontology) terms (7): PAPPA, PRSS8, NT-proBNP, FS, CPB1, CA5A, DLK-1. Catabolic process (8): TIMP4, CTSD, PRELP, MMP-3, CTSZ, TNF-R2, AP-N, LEP. Cell adhesion (8): SLAMF7, IL-4RA, Ep-CAM, IGFBP-2, LEP, IL1RL2, CDH5, CD93. MAPK cascade (8): OPG, GDF-15, REN, CHI3L1, GDF-2, FGF21, GH, LEP. Inflammatory response (12): OPG, IL-4RA, ACE2, TNFRSF10A, PTX3, IL-27, ST2, CHI3L1, LEP, FABP4, IL1RL2, RARRES2.

Out of 182 protein biomarkers included, 39 were associated with ASM (FDR adjusted *P* < 0.05) in the total sample, and only these are shown in Fig. 1. Twenty-nine of these biomarkers were associated with lower ASM in the total sample, most of which are related to the inflammatory response, MAPK cascade, catabolic process, immune response, proteolysis, and other biological processes, as shown in Fig. 1.

The 10 biomarkers that were associated with higher ASM are mostly related to the inflammatory response, cell adhesion, blood vessel morphogenesis and angiogenesis, as shown in Fig. 1. All tested linear regression models for ASM in the total sample and corresponding sex-interaction *P* values are shown in Additional Table 2. The specific biomarkers related to each biological process are listed in the footnote of Fig. 1.

#### Sex-stratified

**Figure 2:**
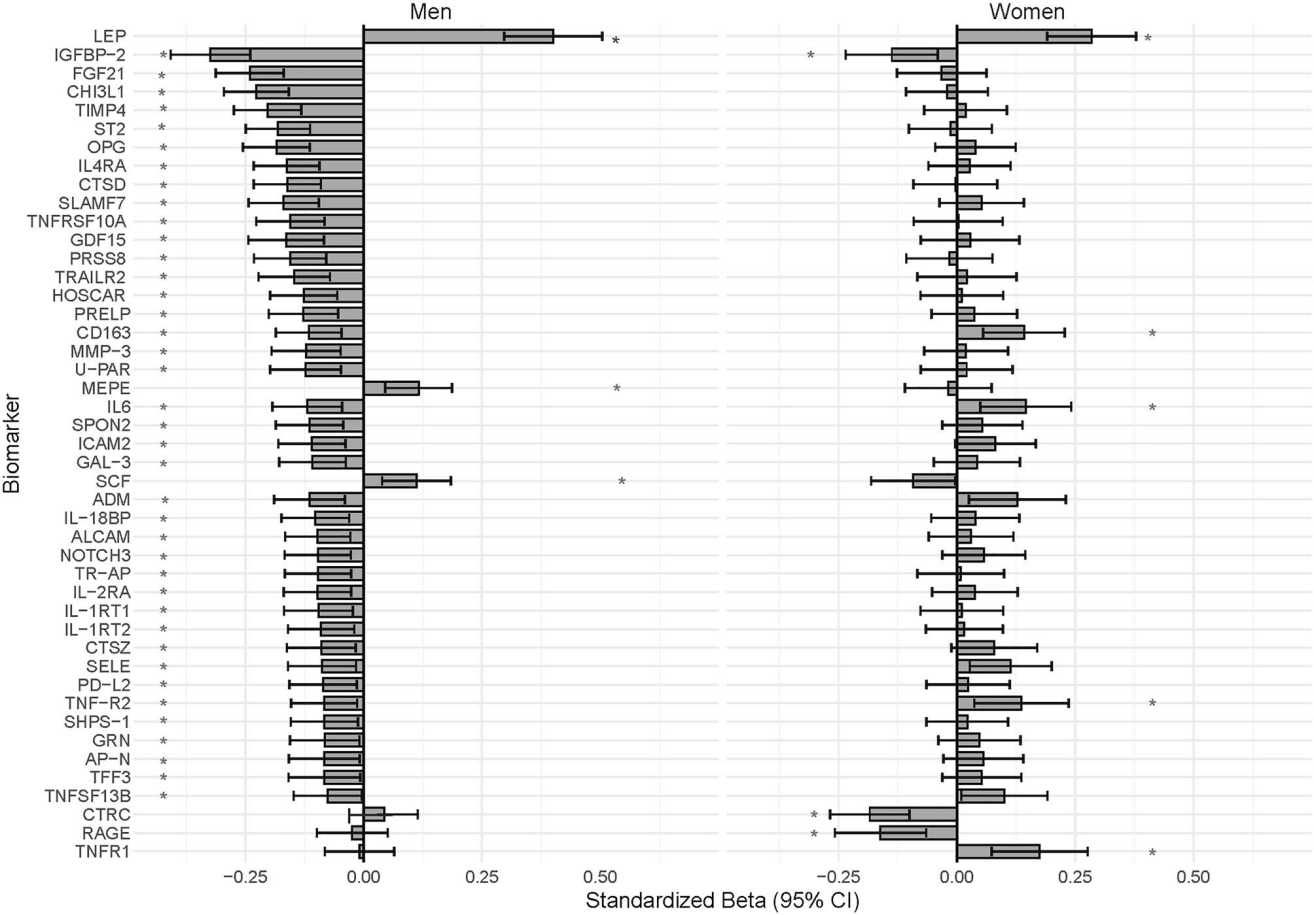
Sex-stratified associations between biomarkers and ASM. Only the 45 biomarkers that showed significant sex interaction and were significantly associated with ASM in at least one of the sex groups are shown. Linear regression models were adjusted for age, height, sex, smoking, alcohol, HFIAS total score, total physical activity (ENMO), visceral adipose tissue, and HIV status. Menopause status was included as an additional confounder in women. *False-discovery rate adjusted *P* value < 0.05; Standardized Beta: Standardized Beta coefficient; 95% CI 95% Confidence Intervals. Full names of all biomarkers are listed in Additional Table 1.

**Figure 3:**
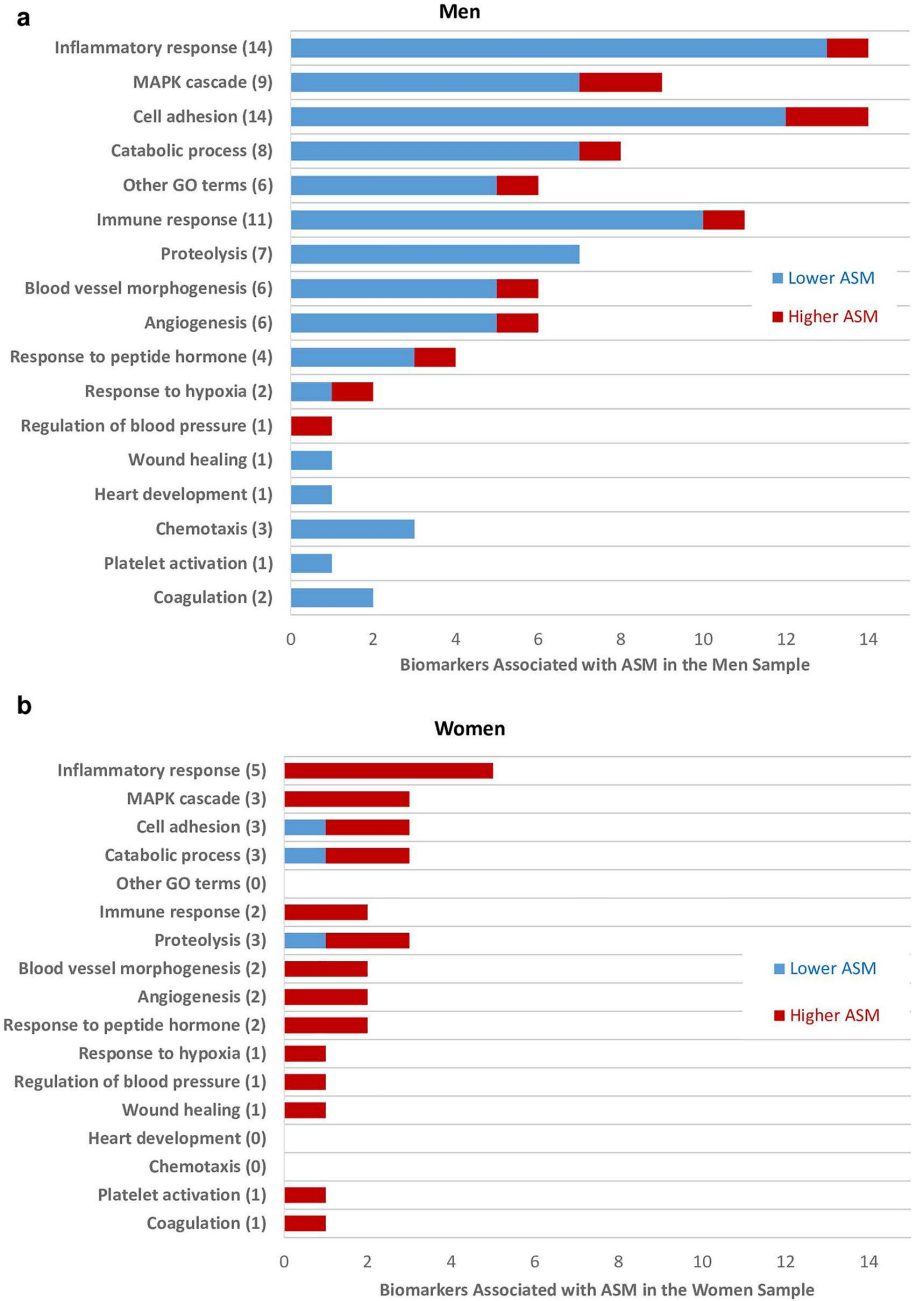
Biological processes related to biomarkers that were associated with ASM. The numbers in brackets represent total biomarkers associated with the outcome and related to the respective biological process. The list of biomarkers associated with the biological processes are based on the OLINK website (www.olink.com/products-services/target). *FOR MEN ONLY (a);* Coagulation (2): U-PAR, IL6. Platelet activation (1): IL6. Chemotaxis (3): U-PAR, GAL-3, ALCAM. Heart development (1): ADM. Wound healing (1): IL6. Regulation of blood pressure (1): LEP. Response to hypoxia (2): ADM, LEP. Response to peptide hormone (4): TIMP4, IL6, ADM, LEP. Angiogenesis (6): CHI3L1, IL6, ADM, NOTCH3, AP-N, LEP. Blood vessel morphogenesis (6): CHI3L1, IL6, ADM, NOTCH3, AP-N, LEP. Proteolysis (7): TIMP4, TNFRSF10A, TRAIL-R2, MMP-3, IL6, CTSZ, TNF-R2. Immune response (11): IL-4RA, SLAMF7, TNFRSF10A, TRAIL-R2, HOSCAR, IL6, SPON2, ADM, PD-L2, TNFRSF13B, LEP. Other GO (Gene Ontology) terms (6): PRSS8, IL-18BP, IL-1RT2, GRN, TFF3, MEPE. Catabolic process (8): TIMP4, CTSD, PRELP, MMP-3, CTSZ, TNF-R2, AP-N, LEP. Cell adhesion (14): IGFBP-2, IL-4RA, SLAMF7, IL6, SPON2, ICAM-2, GAL-3, ALCAM, TR-AP, IL2-RA, SELE, SHPS-1, LEP, SCF. MAPK cascade (9): CHI3L1, FGF21, OPG, GDF-15, IL6, IL2-RA, TNF-R2, LEP, SCF. Inflammatory response (14): CHI3L1, ST2, OPG, IL-4RA, TNFRSF10A, TRAIL-R2, CD163, IL6, TR-AP, IL2-RA, IL-1RT1, SELE, TNF-R2, LEP. *FOR WOMEN ONLY (b);* Coagulation (1): IL6. Platelet activation (1): IL6. Wound healing (1): IL6. Regulation of blood pressure (1): LEP. Response to hypoxia (1): LEP. Response to peptide hormone (2): IL6, LEP. Angiogenesis (2): IL6, LEP. Blood vessel morphogenesis (2): IL6, LEP. Proteolysis (3): CTRC, IL6, TNF-R2. Immune response (2): IL6, LEP. Catabolic process (3): RAGE, LEP, TNF-R2. Cell adhesion (3): IGFBP-2, LEP, IL6. MAPK cascade (3): TNF-R2, LEP, IL6. Inflammatory response (5): CD163, IL6, TNF-R1, TNF-R2, LEP.

Fifty-two biomarkers showed significant sex interactions for ASM (Additional Table 2) and were stratified by sex, all of which are presented in Additional Table 3. From these sex-stratified models, 35 biomarkers were associated with lower ASM in men only (Fig. 2). Most of these biomarkers are related to the inflammatory response, MAPK cascade, cell adhesion, catabolic process, immune response, proteolysis, blood vessel morphogenesis and angiogenesis, and other GO terms, as shown in Fig. 3. Biomarkers related to each biological process are listed in the footnote of Fig. 3. Conversely, SCF and MEPE, which are related to the MAPK cascade and other GO processes, respectively, were associated with higher ASM in men only (Figs. 2, 3).

Three biomarkers, CD163, TNF-R2 and IL6 were associated with lower ASM in men but higher ASM in women (Figs. 2, 3). While these three biomarkers are related to the inflammatory response, TNF-R2 is also related to the catabolic process, MAPK cascade and proteolysis. In contrast, IL6 is related to several other biological pathways including angiogenesis, blood vessel morphogenesis, cell adhesion, coagulation, immune response, MAPK cascade, platelet activation, proteolysis, response to peptide hormone and wound healing.

Further, IGFBP-2 was associated with lower ASM, while LEP was associated with higher ASM, in both men and women (Fig. 2). While these two biomarkers are both related to cell adhesion, LEP is also related to angiogenesis, blood vessel morphogenesis, catabolic process, immune response, inflammatory response, MAPK cascade, regulation of blood pressure, response to hypoxia and response to peptide hormone (Fig. 3).

Finally, CTRC (proteolysis) and RAGE (catabolic process, cell adhesion, immune and inflammatory responses) were associated with lower ASM, while TNF-R1 (inflammatory response) was associated with higher ASM in women only (Figs. 2, 3).

#### Normal versus low ASM

**Figure 4:**
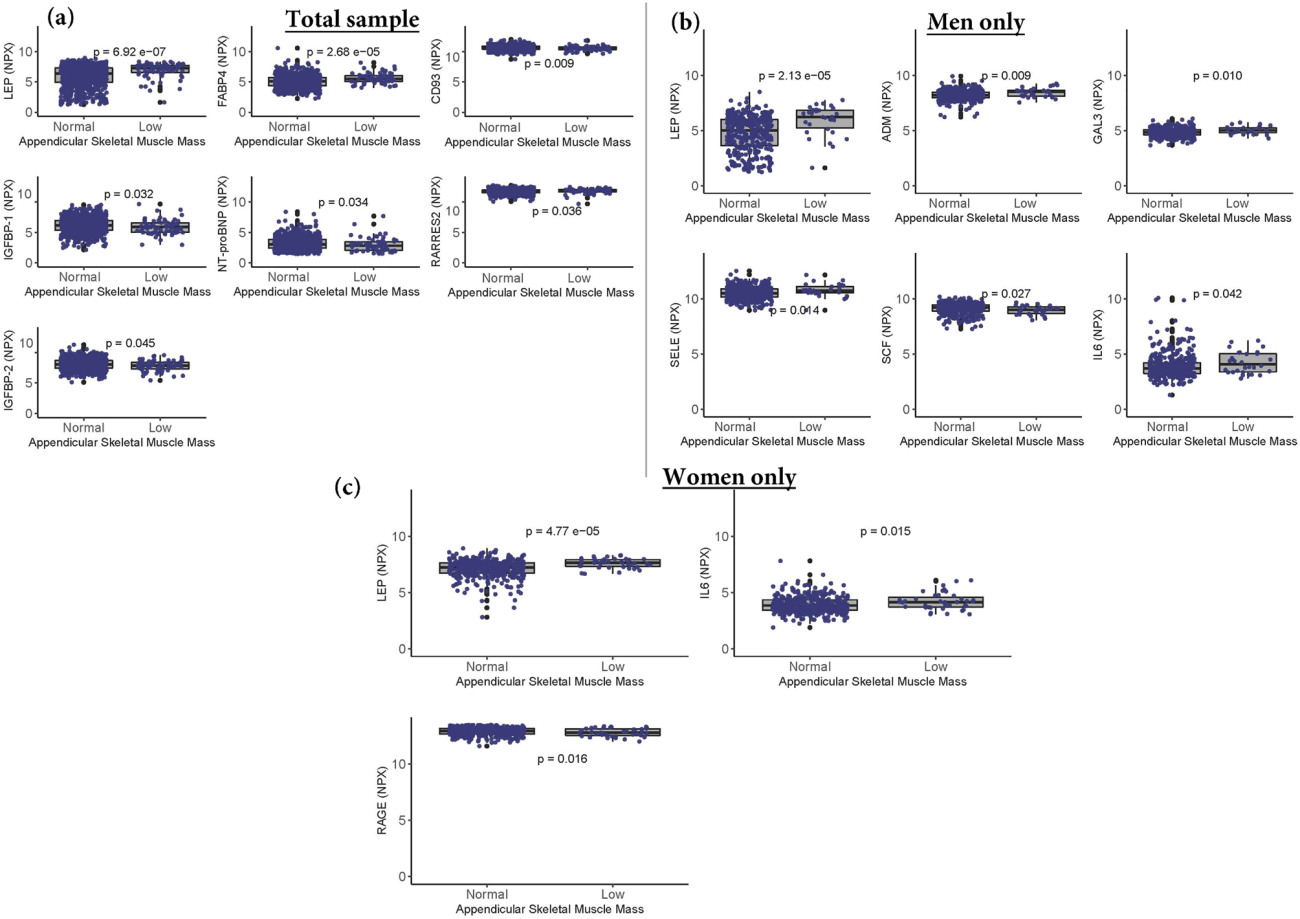
Comparison of biomarkers between participants with normal and low ASM. The Wilcoxon rank-sum test was used to compare groups. The box and whisker plots represent medians and interquartile ranges. NPX: Normalized protein expression; p: *P* value. Full names of all biomarkers are listed in Additional Table 1.

Biomarkers that were associated with ASM in the linear regression models were compared between participants with normal and low ASM, and these are presented in Additional Table 4 (n = 39 models for the total sample), Table 5 (n = 42 models for men only), and Table 6 (n = 8 models for women only). Out of the 39 biomarkers in the total sample, four biomarkers (CD93, IGFBP-1, IGFBP-2 and NT-proBNP) were lower and three biomarkers (FABP4, LEP, and RARRES2) were higher in the low ASM group than the normal group (Fig. 4). In the sample comprising men only, out of the 42 biomarkers (Additional Table 5), ADM, GAL-3, IL6, LEP and SELE were higher and SCF was lower in the low ASM than the normal group (Fig. 4). In the sample comprising women only, out of eight biomarkers (Additional Table 6), IL6 and LEP were higher, while only RAGE was lower, in the low ASM than the normal group. Only biomarkers that were different between the normal and low ASM groups are presented in Fig. 4.

### Associations with HGS

#### Total sample

**Figure 5:**
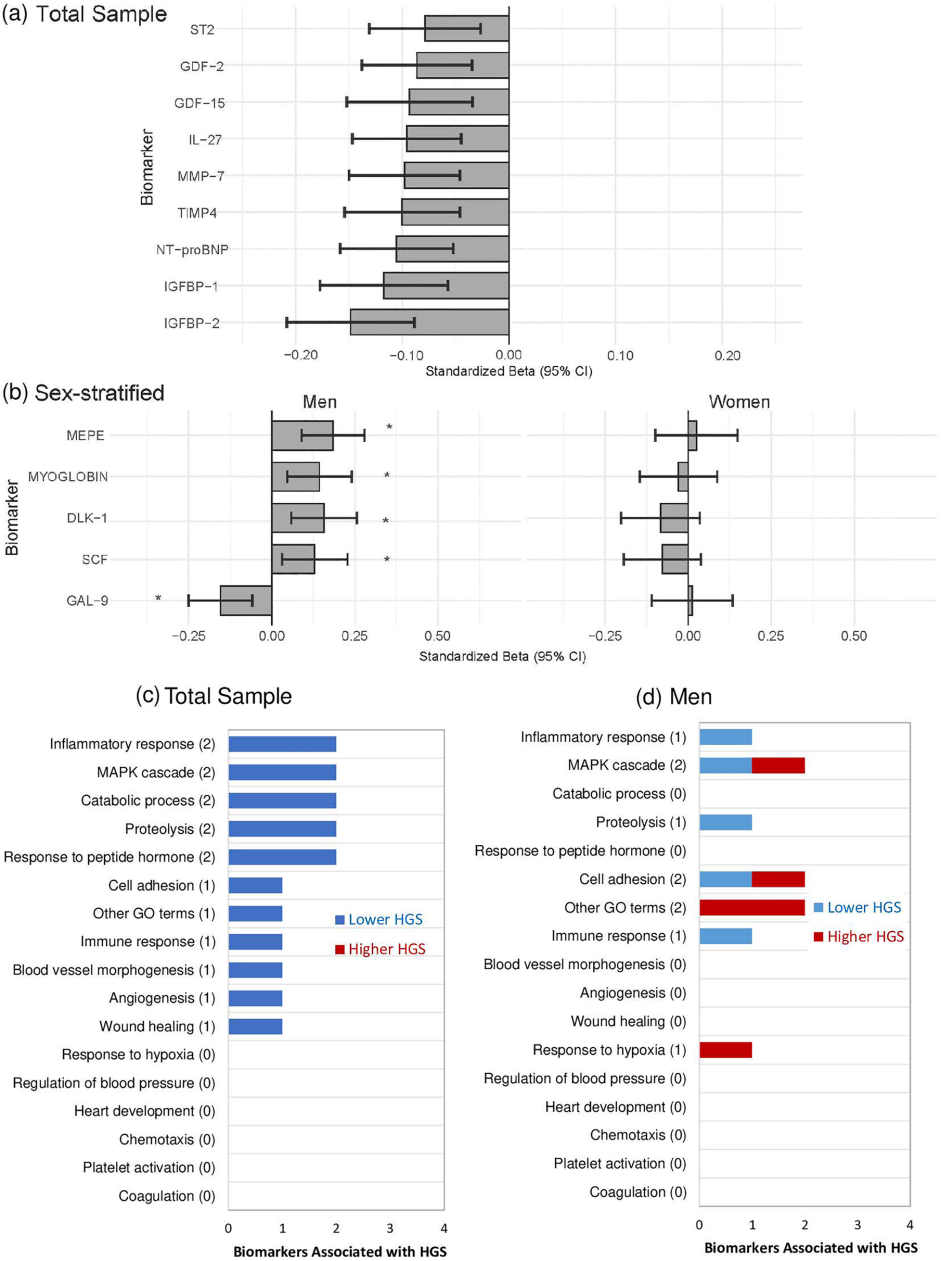
Associations between biomarkers and HGS. Linear regression models (**a**,**b**) were adjusted for age, height, sex, smoking, alcohol, HFIAS total score, total physical activity (ENMO), visceral adipose tissue, and HIV status. Menopause status was included as an additional confounder in women. The numbers in brackets represent total biomarkers associated with the outcome and related to the respective biological process. The list of biomarkers associated with the biological processes are based on the OLINK website (www.olink.com/products-services/target). *False-discovery rate adjusted *P* value < 0.05; Standardized Beta: Standardized Beta coefficient; 95% CI 95% Confidence Intervals; GO: Gene Ontology. Full names of all biomarkers are listed in Additional Table 1.

Of 182 biomarkers included, nine were associated with lower HGS and none were associated with higher HGS in the total sample (Fig. 5a). These included biomarkers related to the inflammatory response (ST2, IL-27), MAPK cascade (GDF-2, GDF-15), catabolic process and proteolysis (MMP-7, TIMP4), response to peptide hormone (TIMP-4, IGFBP-1), cell adhesion (IGFBP-2), immune response (IL-27), blood vessel morphogenesis and angiogenesis (GDF-2), and wound healing (IGFBP-1) (Fig. 5c). All tested linear regression models for HGS in the total sample and corresponding sex-interaction *P* values are shown in Additional Table 7.

#### Sex-stratified

Of 182 tested biomarkers, DLK-1, GAL-9, MEPE, MYOGLOBIN, SCF, TR showed significant sex interactions for HGS (Additional Table 7) and were stratified by sex. From these six biomarkers, TR was not associated with HGS in either men or women, while four biomarkers were associated with higher HGS and one with lower HGS in the men only (Fig. 5b,d). The four biomarkers associated with higher HGS are related to different pathways specifically around the MAPK cascade and cell adhesion (SCF), response to hypoxia (MYOGLOBIN), and other GO terms (MEPE and DLK-1) (Fig. 5b,d). The biomarker, GAL-9, which was associated with lower HGS in the men only is related to cell adhesion, immune response, inflammatory response, MAPK cascade and proteolysis (Fig. 5b,d). No significant sex-stratified associations were shown in the women.

#### Normal versus low HGS

**Figure 6:**
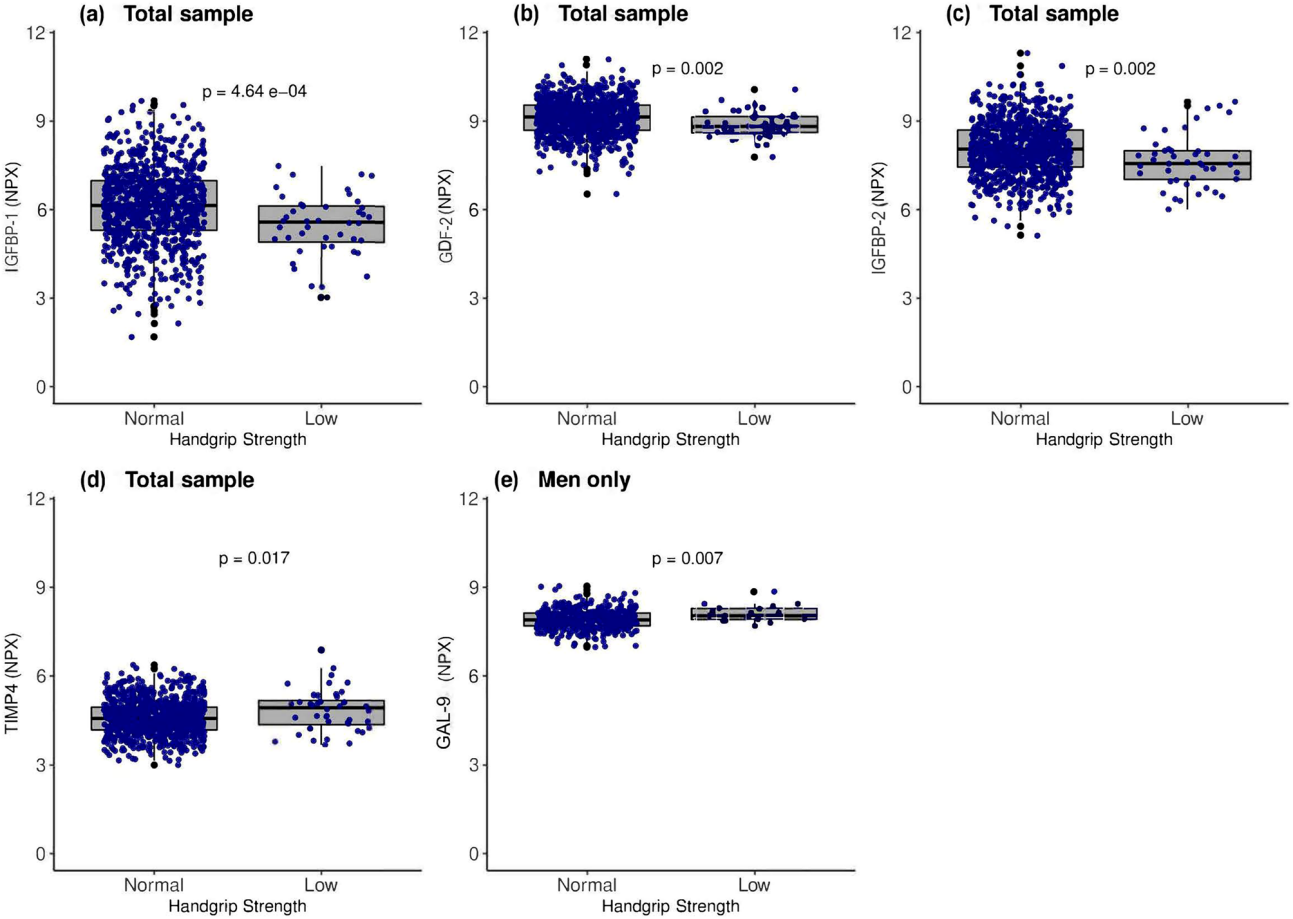
A comparison of selected biomarkers between participants with normal and low HGS. The Wilcoxon rank-sum test was used to compare groups. The box and whisker plots represent medians and interquartile ranges. NPX: Normalized protein expression; p: *P* value. Full names of all biomarkers are listed in Additional Table 1.

Biomarkers that showed significant associations with HGS in the linear regression models were compared between participants with normal and low HGS. Of the nine biomarkers that were compared in the total sample (Additional Table 9), GDF-2, IGFBP-1, and IGFBP-2 were lower in the low HGS group compared to the normal group (Fig. 6). In contrast, of the five biomarkers that were significant in the men only (Additional Table 10), only GAL-9 was higher in the low HGS group than the normal group (Fig. 6). Only biomarkers that were different between the normal and low HGS groups are shown in Fig. 6.

### Associations with both ASM and HGS

**Figure 7:**
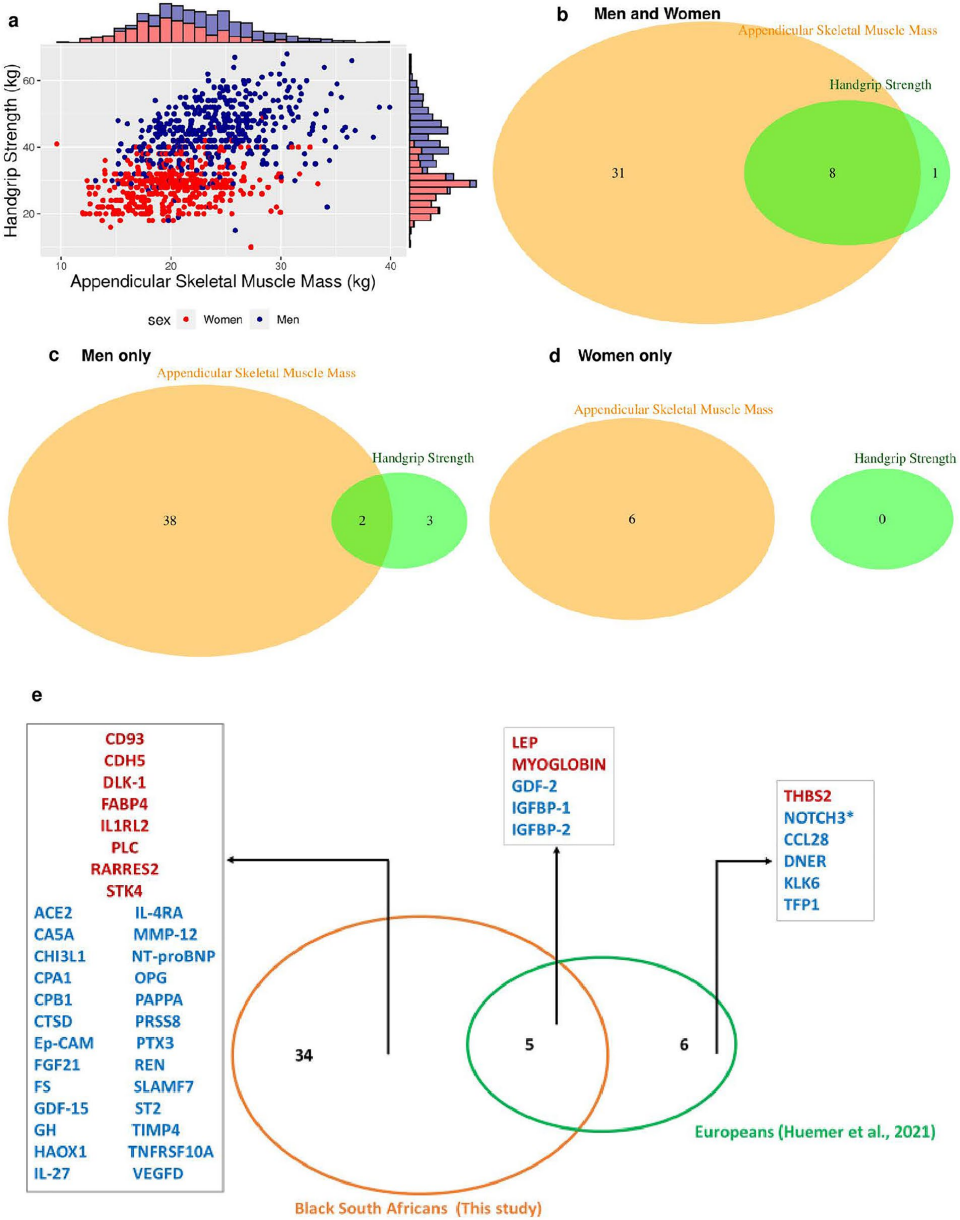
Relationship between ASM and HGS and biomarker comparison with European findings^19^. Biomarkers in red were associated with higher ASM and biomarkers in blue were associated with lower ASM. *Associated with lower ASM in Europeans but with higher ASM in black South African men only (**e**).

There was a linear relationship between ASM and HGS in both men and women (Spearman’s rho = 0.377 and 0.263, respectively; Fig. 7a). The numbers of biomarkers shown in Fig. 7b–d were determined by comparing significant biomarkers (FDR-adjusted *P* < 0.05) from all linear regression models of this study.

In the total sample, eight biomarkers (GDF-15, GDF-2, IGFBP-1, IGFBP-2, IL-27, NT-proBNP, ST2, TIMP4) were associated with both ASM and HGS, 31 were associated with ASM only, and only MMP-7 was associated with HGS only (Fig. 7b). In men, 38 biomarkers were associated with ASM only, three (GAL-9, DLK-1, MYOGLOBIN) were associated with HGS only, and two (MEPE, SCF) with both outcomes (Fig. 7c). In women, six biomarkers (CD163, IL6, TNF-R2, CTRC, RAGE, TNF-R1) were associated with ASM and none with HGS (Fig. 7d).

## Discussion

The present study used targeted proteomics to identify a wide range of circulating biomarkers that were associated with ASM and HGS in middle-aged Black South African men and women. Importantly, most biomarkers were associated with ASM and not HGS, with only 8 of the 184 biomarkers being associated with both. Furthermore, many of the associations were sex-specific, with the majority being observed in men. These findings add new knowledge to our understanding of sarcopenia, as previous proteomic studies did not investigate sex-interactions and only considered sarcopenia or ASM as the outcome of interest, assuming that biomarkers of quantity also predict muscle function^16–19^.

The identified biomarkers are related to a range of biological pathways, reflecting the complexity of metabolic mechanisms associated with ASM and/or HGS. Our finding that only a few biomarkers were associated with both ASM and HGS is in accordance with previous findings that other biological factors, especially the nervous system, determine muscle function/strength and that it does not only depend on the quantity of muscle^27^. Genome-wide association studies have demonstrated that several variants associated with HGS were at genes involved in neuro-developmental disorders/brain function^28,29^. In our study only one biomarker, MMP-7 which plays a role in the breakdown of extracellular matrix, was associated with HGS and not ASM in the total sample^30^. Furthermore, several factors, including hand circumference, forearm girth, and psychological health status, may influence HGS but not ASM^31^.

### Biomarkers associated with both ASM and HGS

We found that only eight biomarkers, IGFBP-1, IGFBP-2, GDF-15, GDF-2, IL-27, ST2, NT-proBNP, TIMP4, were associated with both ASM and HGS in the total sample. Although these biomarkers have not been previously associated with HGS, most of them were identified within the biological pathways of muscle growth and loss, and may influence muscle function via quantity. For example, IGFBP-1 and 2 are known to bind and regulate the bioavailability of insulin-like growth factors which play a role in muscle growth and regeneration^9^. Similar to our findings, IGFBP-1 was previously associated with lower ASM in older Swedish women^32^. Growth-differentiation factors (GDF-2 and GDF-15) stimulate an increase in phosphorylation of the MAPK cascade, which can also be stimulated by other factors including inflammation, and subsequently continues with a series of activating phosphorylation stages to regulate myogenesis^33^. Inflammatory biomarkers, such as IL-27 and ST2, generally associate with lower ASM, and their effects on muscle wasting may be mediated by the Nuclear factor kappa B transcription factor, as its activation leads to degradation of specific proteins within muscle^9^. NT-proBNP is an N-terminal prohormone that is cleaved from the brain natriuretic peptide (BNP) precursor to release BNP and is used in the diagnosis of acute congestive heart failure^19^. Our observation that NT-proBNP was lower in individuals with low ASM compared to the normal group, was in accordance with the previous study from Germany. In that study, NT-proBNP levels were associated with a decrease in ASM after 14 years^19^. TIMP4 is acknowledged as a modulator of MMP9 and known for its role in cardiac stem cell differentiation and myocardial construction. While, this biomarker is highly expressed in the muscle and adipose tissue, its role in these tissues remain elusive^34^.

### Comparison with findings from Europeans

A recent proteomic study of ASM and body fat mass index (measured using bioelectrical impedance) in 1478 men and women from Germany (aged 55–74 years) investigated 233 biomarkers from the OLINK’s CVD II, CVD III and inflammatory panels^19^. Five (GDF-2, IGFBP-1, IGFBP-2, LEP, and MYOGLOBIN) of the nine biomarkers measured using the CVD II and III panels in the German study, that were associated with ASM, were replicated in our study (Fig. 7e)^19^. Although NOTCH3 was positively associated with ASM in that European study, this biomarker was negatively associated with ASM in men only in the present study.

Comparison of our findings with those from Europeans should still be viewed with caution as ethnic differences in body fat distribution and biomarker profiles are known between Black South Africans and their European counterparts^35^. Studies have shown that Black South African women present with hyperinsulinemia resulting from increased insulin secretion and reduced hepatic insulin clearance compared to their counterparts^35^. Likewise, women of African ancestry may have higher skeletal muscle and body fat mass and inflammatory biomarker levels compared to European ancestry counterparts^36,37^. Besides ethnic differences in the observed associations, study design may have contributed to the discrepancies between the European findings and our observations. In the German study, several confounders that were included in the analysis were cardiometabolic risk factors (high-density lipoprotein, triglycerides, glycated hemoglobin, and glomerular filtration rate), which are likely to be involved in the pathophysiology of sarcopenia^9^. Adjusting for these variables may have excluded other potential biological pathways. Moreover, the German study did not adjust for adiposity in their analyses, possibly because adiposity measures were correlated with one of their outcomes (body fat mass index). Hence, many biomarkers were likely missed by not adjusting for adiposity, which is a well-known confounder in complex diseases^20^. Consistent with the confounding effects of adiposity, in the German study, the direction of association between some of the biomarkers (LEP, THBS2 and GDF-2) and ASM changed when body fat mass was included in the statistical models^19^.

### Sex-specific associations

Another novel finding of the present study were sex-specific associations with ASM and HGS, of which most were specific to men. Two biomarkers, MEPE and SCF were associated with both higher ASM and HGS in men only in the present study. While these two biomarkers are known for their involvement in bone growth and regulation, MEPE regulates mineralization and is involved in renal phosphate excretion, while SCF acts as a cytokine and is involved in hematopoiesis, spermatogenesis, and melanogenesis^38^. We are not aware of any study that has reported similar associations and although these biomarkers were included in the German study for association with ASM, they were likely missed as a result of not testing sex-interactions^19^.

Certainly, sex differences in the expression levels of CVD biomarkers are known^39^. However, this is the first study to demonstrate that associations between these biomarkers and components of sarcopenia differ by sex. Sex-specific associations in risk factors of complex traits are well acknowledged, and thought to be primarily driven by differences in body fat distribution and sex hormones, notably differences in VAT and estrogen/testosterone levels^40^. Our findings were adjusted for VAT but further studies including measurements of sex hormones are of major interest to improve the understanding of the observed sex-specific associations.

### Study limitations and strengths

Our study has some limitations. The generalizability of our findings to other ethnicities may be limited as ethnic differences in complex phenotypes are known. Furthermore, the targeted proteomics approach may have excluded non-targeted biomarkers potentially relevant to ASM and HGS. Regardless of these limitations, this is the largest study to investigate biomarkers of ASM and HGS in black South Africans and the first study to investigate sex-specific relationships in biomarkers of sarcopenia components.

## Conclusions

We found that most biomarkers of ASM were not associated with HGS, and that the relationships between biomarkers and components of sarcopenia displayed sex-specificity in middle-aged Black South Africans. These findings support the hypothesis that biomarkers associated with ASM may not necessarily predict HGS. Therefore, future proteomic studies of sarcopenia components should ensure that both ASM and HGS are individually examined. Furthermore, previous proteomic studies of sarcopenia-related components may have missed sex-specific relationships by not testing for sex-interactions. Our findings highlight the need to consider sex-specificity in the pathophysiology of sarcopenia for development of sex-specific treatment and diagnostic methods.

## Supplementary Information

Supplementary Information 1.

Supplementary Information 2.

Supplementary Information 3.

Supplementary Information 4.

Supplementary Information 5.

Supplementary Information 6.

Supplementary Information 7.

Supplementary Information 8.

Supplementary Information 9.

Supplementary Information 10.

Supplementary Information 11.

## Data Availability

The datasets generated and/or analyzed during the current study are not publicly available as the study has only just been completed and data will only be made publicly available after a 2-year period. However, data can be made available from the corresponding author on reasonable request.

## Abbreviations

ASM: Appendicular skeletal muscle mass
BMI: Body mass index
CVD: Cardiovascular disease
DXA: Dual-energy X-ray absorptiometry
ENMO: Euclidian norm minus one
FDR: False-discovery rate
GO: Gene ontology
HFIAS: Household food insecurity scale
HGS: Handgrip strength
HIV: Human immunodeficiency virus
MAPK: Mitogen-activated protein kinase
MASC: Middle-aged Soweto cohort
M*g*: Milli-*g*
NPX: Normalized protein expression
VAT: Visceral adipose tissue
