# Supplementary figures and images for: Targeted proteomics of appendicular skeletal muscle mass and handgrip strength in black South Africans: a cross-sectional study
Source: Sci Rep. 2022 Jun 9;12:9512. doi: 10.1038/s41598-022-13548-9 (PMC9178538; doi:10.1038/s41598-022-13548-9)

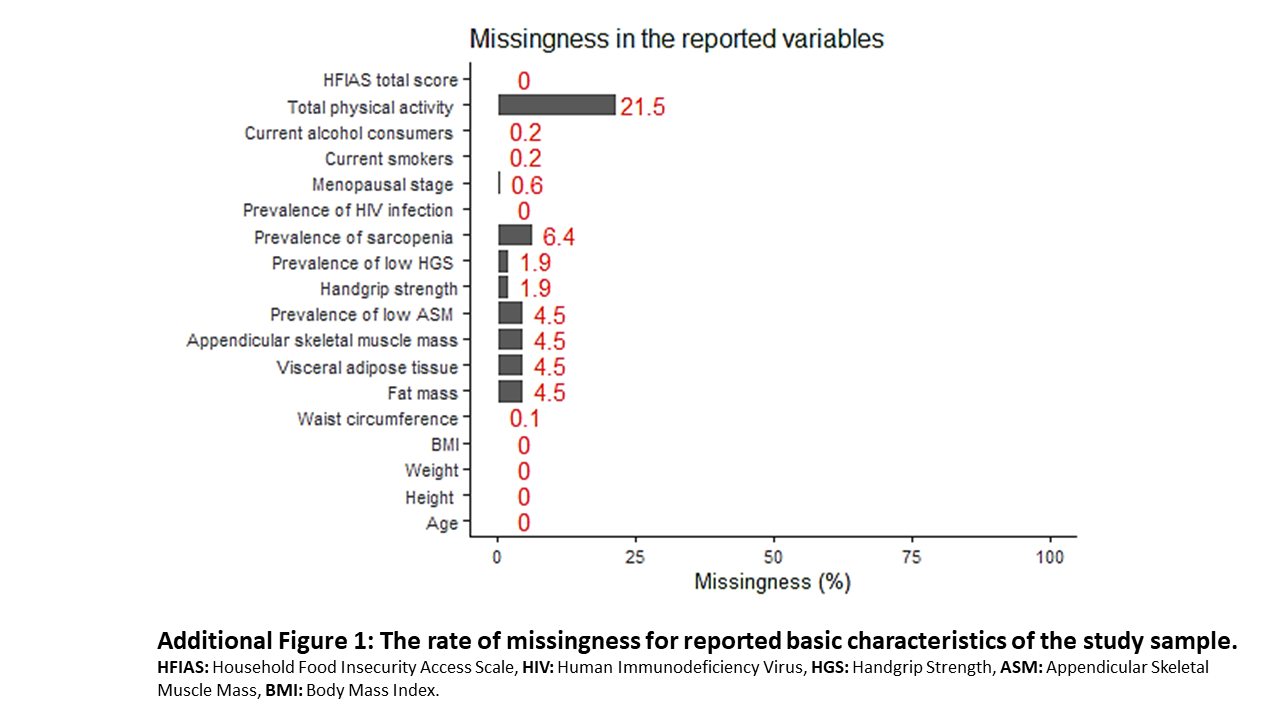

Supplement: Supplementary file 1 — Supplementary Information 1. [file 41598_2022_13548_MOESM1_ESM.tif]
