# Supplementary material for: Targeted proteomics of appendicular skeletal muscle mass and handgrip strength in black South Africans: a cross-sectional study
Source: Sci Rep. 2022 Jun 9;12:9512. doi: 10.1038/s41598-022-13548-9 (PMC9178538; doi:10.1038/s41598-022-13548-9)
Supplement: Supplementary file 2 — Supplementary Information 2. [file 41598_2022_13548_MOESM2_ESM.docx]

**Additional Table 1: Full names of all protein biomarkers included in the present study.**

| **Abbreviation** | **Full Name** | **Related Biological Processes** |
| --- | --- | --- |
| ACE2 | Angiotensin-converting enzyme 2 | Catabolic process, Inflammatory response, Proteolysis, Regulation of blood pressure |
| ADAM-TS13 | A disintegrin and metalloproteinase with thrombospondin motifs 13 | Catabolic process, Cell adhesion, Coagulation, Immune response, Platelet activation, Proteolysis, Wound healing |
| ADM | Adrenomedullin | Angiogenesis, Blood vessel morphogenesis, Heart development, Immune response, Response to hypoxia, Response to peptide hormone |
| AGRP | Agouti-related protein | Response to peptide hormone |
| ALCAM | CD166 antigen | Cell adhesion, Chemotaxis |
| AMBP | Protein AMBP | Catabolic process, Cell adhesion, Immune response, MAPK cascade |
| ANGPT1 | Angiopoietin-1 | Angiogenesis, Blood vessel morphogenesis, Cell adhesion, Immune response, MAPK cascade |
| AP-N | Aminopeptidase N | Angiogenesis, Blood vessel morphogenesis, Catabolic process |
| AXL | Tyrosine-protein kinase receptor UFO | Cell adhesion, Coagulation, Inflammatory response, Platelet activation, Wound healing |
| AZU1 | Azurocidin | Cell adhesion, Chemotaxis, Inflammatory response, Proteolysis |
| BLM HYDROLASE | Bleomycin hydrolase | Proteolysis |
| BMP-6 | Bone morphogenetic protein 6 | Immune response, Inflammatory response, MAPK cascade |
| BOC | Brother of CDO | Catabolic process, Cell adhesion |
| CA5A | Carbonic anhydrase 5A, mitochondrial | Other GO terms |
| CASP-3 | Caspase-3 | Catabolic process, Cell adhesion, Proteolysis, Response to hypoxia, Wound healing |
| CCL15 | C-C motif chemokine 15 | Chemotaxis, Inflammatory response, MAPK cascade |
| CCL16 | C-C motif chemokine 16 | Chemotaxis, Inflammatory response, MAPK cascade |
| CCL17 | C-C motif chemokine 17 | Immune response, Inflammatory response, MAPK cascade |
| CCL24 | C-C motif chemokine 24 | Angiogenesis, Blood vessel morphogenesis, Chemotaxis, Inflammatory response, MAPK cascade |
| CCL3 | C-C motif chemokine 3 | Immune response, Inflammatory response, MAPK cascade |
| CD163 | Scavenger receptor cysteine-rich type 1 protein M130 | Inflammatory response |
| CD4 | T-cell surface glycoprotein CD4 | Cell adhesion, Immune response |
| CD40-L | CD40 ligand | Cell adhesion, Coagulation, Immune response, Inflammatory response, MAPK cascade, Platelet activation, Wound healing |
| CD84 | SLAM family member 5 | Cell adhesion, Immune response |
| CD93 | Complement component C1q receptor | Catabolic process, Cell adhesion |
| CDH5 | Cadherin-5 | Cell adhesion |
| CEACAM8 | Carcinoembryonic antigenrelated cell adhesion molecule 8 | Immune response |
| CHI3L1 | Chitinase-3-like protein 1 | Angiogenesis, Blood vessel morphogenesis, Inflammatory response, MAPK cascade |
| CHIT1 | Chitotriosidase-1 | Catabolic process |
| CNTN1 | Contactin-1 | Cell adhesion |
| COL1A1 | Collagen alpha-1(I) chain | Catabolic process, Cell adhesion, Coagulation, Platelet activation, Response to peptide hormone, Wound healing |
| CPA1 | Carboxypeptidase A1 | Proteolysis |
| CPB1 | Carboxypeptidase B | Other GO terms |
| CSTB | Cystatin-B | Proteolysis |
| CTRC | Chymotrypsin C | Proteolysis |
| CTSD | Cathepsin D | Catabolic process |
| CTSL1 | Cathepsin L1 | Catabolic process, Immune response, Proteolysis |
| CTSZ | Cathepsin Z | Catabolic process, Proteolysis |
| CXCL1 | C-X-C motif chemokine 1 | Immune response, Inflammatory response |
| CXCL16 | C-X-C motif chemokine 16 | Chemotaxis |
| DCN | Decorin | Angiogenesis, Blood vessel morphogenesis, Catabolic process, Wound healing |
| DECR1 | 2,4-dienoyl-CoA reductase, mitochondrial | Catabolic process |
| DKK-1 | Dickkopf-related protein 1 | Heart development |
| DLK-1 | Protein delta homolog 1 | Other GO terms |
| EGFR | Epidermal growth factor receptor | Catabolic process, Cell adhesion, MAPK cascade |
| Ep-CAM | Epithelial cell adhesion molecule | Cell adhesion |
| EPHB4 | Ephrin type-B receptor 4 | Angiogenesis, Blood vessel morphogenesis, Cell adhesion |
| FABP2 | Fatty acid-binding protein, intestinal | Catabolic process |
| FABP4 | Fatty acid-binding protein, adipocyte | Catabolic process, Inflammatory response |
| FAS | Tumor necrosis factor receptor superfamily member 6 | Cell adhesion, Inflammatory response, MAPK cascade, Proteolysis, Response to hypoxia, Response to peptide hormone |
| FGF21 | Fibroblast growth factor 21 | MAPK cascade |
| FGF-23 | Fibroblast growth factor 23 | Catabolic process, MAPK cascade |
| FS | Follistatin | Other GO terms |
| GAL-3 | Galectin-3 | Cell adhesion, Chemotaxis |
| GAL-4 | Galectin-4 | Cell adhesion |
| GAL-9 | Galectin-9 | Cell adhesion, Immune response, Inflammatory response, MAPK cascade, Proteolysis |
| GDF-15 | Growth/differentiation factor 15 | MAPK cascade |
| GDF-2 | Growth/differentiation factor 2 | Angiogenesis, Blood vessel morphogenesis, MAPK cascade |
| GH | Growth hormone | MAPK cascade, Response to peptide hormone |
| GIF | Gastric intrinsic factor | Other GO terms |
| GLO1 | Lactoylglutathione lyase | Other GO terms |
| GP6 | Platelet glycoprotein VI | Coagulation, Platelet activation, Wound healing |
| GRN | Granulins | Other GO terms |
| GT | Gastrotropin | Catabolic process |
| HAOX1 | Hydroxyacid oxidase 1 | Catabolic process |
| HB-EGF | Proheparin-binding EGF-like growth factor | MAPK cascade, Wound healing |
| HO-1 | Heme oxygenase 1 | Angiogenesis, Blood vessel morphogenesis, Catabolic process, Immune response, Inflammatory response, Regulation of blood pressure, Response to hypoxia, Wound healing |
| HOSCAR | Osteoclast-associated immunoglobulin-like receptor | Immune response |
| HSP 27 | Heat shock 27 kDa protein | Angiogenesis, Blood vessel morphogenesis, Catabolic process, Cell adhesion, Coagulation, Platelet activation, Wound healing |
| ICAM-2 | Intercellular adhesion molecule 2 | Cell adhesion |
| IDUA | Alpha-L-iduronidase | Catabolic process |
| IGFBP-1 | Insulin-like growth factor-binding protein 1 | Response to peptide hormone, Wound healing |
| IGFBP-2 | Insulin-like growth factor-binding protein 2 | Cell adhesion |
| IGFBP-7 | Insulin-like growth factor-binding protein 7 | Cell adhesion |
| IgG Fc receptor II-b | Low affinity immunoglobulin gamma Fc region receptor II-b | Immune response |
| IL16 | Interleukin-6 | Immune response |
| IL-17D | Interleukin-17D | Inflammatory response |
| IL17RA | Interleukin-17 receptor A | Chemotaxis, Inflammatory response |
| IL-18 | Interleukin-18 | Angiogenesis, Blood vessel morphogenesis, Cell adhesion, Immune response, Inflammatory response, MAPK cascade |
| IL-18BP | Interleukin-18-binding protein | Other GO terms |
| IL-1RA | Interleukin-1 receptor antagonist protein | Cell adhesion, Inflammatory response |
| IL1RL2 | Interleukin-1 receptor-like 2 | Cell adhesion, Immune response, Inflammatory response |
| IL-1RT1 | Interleukin-1 receptor type 1 | Inflammatory response |
| IL-1RT2 | Interleukin-1 receptor type 2 | Other GO terms |
| IL-27 | Interleukin-27 | Immune response, Inflammatory response |
| IL2-RA | Interleukin-2 receptor subunit alpha | Cell adhesion, Inflammatory response, MAPK cascade |
| IL-4RA | Interleukin-4 receptor subunit alpha | Cell adhesion, Immune response, Inflammatory response |
| IL6 | Interleukin-6 receptor subunit alpha | Angiogenesis, Blood vessel morphogenesis, Cell adhesion, Coagulation, Immune response, Inflammatory response, MAPK cascade, Platelet activation, Proteolysis, Response to peptide hormone, Wound healing |
| IL-6RA | Pro-interleukin-16 | Chemotaxis, Inflammatory response, MAPK cascade |
| ITGB1BP2 | Melusin | Other GO terms |
| ITGB2 | Integrin beta-2 | Angiogenesis, Blood vessel morphogenesis, Cell adhesion, Chemotaxis, Inflammatory response |
| JAM-A | Junctional adhesion molecule A | Inflammatory response |
| KIM1 | Kidney Injury Molecule | Other GO terms |
| KLK6 | Kallikrein-6 | Catabolic process, Proteolysis, Wound healing |
| LDL receptor | Low-density lipoprotein receptor | Catabolic process |
| LEP | Leptin | Angiogenesis, Blood vessel morphogenesis, Catabolic process, Cell adhesion, Immune response, Inflammatory response, MAPK cascade, Regulation of blood pressure, Response to hypoxia, Response to peptide hormone |
| LOX-1 | Lectin-like oxidized LDL receptor 1 | Cell adhesion, Inflammatory response, Proteolysis |
| LPL | Lipoprotein lipase | Catabolic process, Inflammatory response |
| LTBR | Lymphotoxin-beta receptor | Inflammatory response, MAPK cascade |
| MARCO | Macrophage receptor MARCO | Immune response |
| MCP-1 | Monocyte chemotactic protein 1 | Angiogenesis, Blood vessel morphogenesis, Cell adhesion, Chemotaxis, Inflammatory response, MAPK cascade, Response to hypoxia, Response to peptide hormone |
| MEPE | Matrix extracellular phosphoglycoprotein | Other GO terms |
| MERTK | Tyrosine-protein kinase Mer | Cell adhesion, Coagulation, Platelet activation, Wound healing |
| MMP-12 | Matrix metalloproteinase-12 | Catabolic process, Proteolysis, Wound healing |
| MMP-2 | Matrix metalloproteinase-2 | Angiogenesis, Blood vessel morphogenesis, Catabolic process, Proteolysis, Response to hypoxia |
| MMP-3 | Matrix metalloproteinase-3 | Catabolic process, Proteolysis |
| MMP-7 | Matrix metalloproteinase-7 | Catabolic process, Proteolysis |
| MMP-9 | Matrix metalloproteinase-9 | Catabolic process, Proteolysis |
| MPO | Myeloperoxidase | Catabolic process |
| MYOGLOBIN | Myoglobin | Response to hypoxia |
| NEMO | NF-kappa-B essential modulator | Immune response, Inflammatory response, MAPK cascade |
| NOTCH3 | Neurogenic locus notch homolog protein 3 | Angiogenesis, Blood vessel morphogenesis |
| NT-proBNP | N-terminal prohormone brain natriuretic peptide | Other GO terms |
| OPG | Osteoprotegerin | Inflammatory response, MAPK cascade |
| OPN | Osteopontin | Cell adhesion, Inflammatory response |
| PAI | Plasminogen activator inhibitor 1 | Angiogenesis, Blood vessel morphogenesis, Cell adhesion, Chemotaxis, Coagulation, Inflammatory response, Proteolysis, Wound healing |
| PAPPA | Pappalysin-1 | Other GO terms |
| PAR-1 | Proteinase-activated receptor 1 | Coagulation, Inflammatory response, MAPK cascade, Platelet activation, Proteolysis, Regulation of blood pressure, Wound healing |
| PARP-1 | Poly [ADP-ribose] polymerase 1 | Proteolysis, Response to peptide hormone |
| PCSK9 | Proprotein convertase subtilisin/kexin type 9 | Catabolic process, Proteolysis, Response to peptide hormone |
| PDGF subunit A | Platelet-derived growth factor subunit A | Angiogenesis, Blood vessel morphogenesis, Chemotaxis, Coagulation, MAPK cascade, Platelet activation, Response to hypoxia, Wound healing |
| PDGF subunit B | Platelet-derived growth factor subunit B | Coagulation, Heart development, MAPK cascade, Platelet activation, Regulation of blood pressure, Response to hypoxia, Response to peptide hormone, Wound healing |
| PD-L2 | Programmed cell death 1 ligand 2 | Immune response |
| PECAM-1 | Platelet endothelial cell adhesion molecule | Cell adhesion |
| PGF | Placenta growth factor | Angiogenesis, Blood vessel morphogenesis, Response to hypoxia |
| PGLYRP1 | Peptidoglycan recognition protein 1 | Catabolic process, Inflammatory response |
| PI3 | Elafin | Other GO terms |
| PIgR | Polymeric immunoglobulin receptor | Immune response |
| PLC | Perlecan | Angiogenesis, Blood vessel morphogenesis, Catabolic process |
| PON3 | Paraoxonase | Catabolic process |
| PRELP | Prolargin | Catabolic process |
| PRSS27 | Serine protease 27 | Other GO terms |
| PRSS8 | Prostasin | Other GO terms |
| PRTN3 | Myeloblastin | Catabolic process, Coagulation, Wound healing |
| PSGL-1 | P-selectin glycoprotein ligand 1 | Cell adhesion |
| PSP-D | Pulmonary surfactant-associated protein D | Cell adhesion, Chemotaxis |
| PTX3 | Pentraxin-related protein PTX3 | Immune response, Inflammatory response |
| RAGE | Receptor for advanced glycosylation end products | Catabolic process, Cell adhesion, Immune response, Inflammatory response |
| RARRES2 | Retinoic acid receptor responder protein 2 | Catabolic process, Chemotaxis, Inflammatory response, Response to peptide hormone |
| REN | Renin | MAPK cascade, Proteolysis, Regulation of blood pressure |
| RETN | Resistin | Response to peptide hormone |
| SCF | Stem cell factor | Cell adhesion, MAPK cascade |
| SCGB3A2 | Secretoglobin family 3A member 2 | Other GO terms |
| SELE | E-selectin | Cell adhesion, Inflammatory response |
| SELP | P-selectin | Cell adhesion, Coagulation, Inflammatory response, Platelet activation, Wound healing |
| SERPINA12 | Serpin A12 | Response to peptide hormone |
| SHPS-1 | Tyrosine-protein phosphatase non-receptor type substrate 1 | Cell adhesion |
| SLAMF7 | SLAM family member 7 | Cell adhesion, Immune response |
| SOD2 | Superoxide dismutase [Mn], mitochondrial | Regulation of blood pressure |
| SORT1 | Sortilin | Response to peptide hormone |
| SPON2 | Spondin-2 | Cell adhesion, Immune response |
| SRC | Proto-oncogene tyrosine-protein kinase Src | Cell adhesion, Coagulation, Immune response, MAPK cascade, Platelet activation, Proteolysis, Response to hypoxia, Response to peptide hormone, Wound healing |
| ST2 | ST2 protein | Inflammatory response |
| STK4 | Serine/threonine-protein kinase 4 | Angiogenesis, Blood vessel morphogenesis, Heart development |
| TF | Tissue factor | Angiogenesis, Blood vessel morphogenesis, Coagulation, Inflammatory response, Proteolysis, Wound healing |
| TFF3 | Trefoil factor 3 | Other GO terms |
| TFPI | Tissue factor pathway inhibitor | Coagulation, Wound healing |
| TGM2 | Protein-glutamine gamma-glutamyltransferase 2 | Cell adhesion |
| THBS2 | Thrombospondin-2 | Angiogenesis, Blood vessel morphogenesis, Cell adhesion |
| THPO | Thrombopoietin | MAPK cascade |
| TIE2 | Angiopoietin-1 receptor | Angiogenesis, Blood vessel morphogenesis, Cell adhesion, Heart development, Inflammatory response, MAPK cascade, Response to hypoxia, Response to peptide hormone |
| TIMP4 | Metalloproteinase inhibitor 4 | Catabolic process, Proteolysis, Response to peptide hormone |
| TLT-2 | Trem-like transcript 2 protein | Cell adhesion |
| TM | Thrombomodulin | Coagulation, Platelet activation, Wound healing |
| TNF-R1 | Tumor necrosis factor receptor 1 | Inflammatory response |
| TNF-R2 | Tumor necrosis factor receptor 2 | Catabolic process, Inflammatory response, MAPK cascade, Proteolysis |
| TNFRSF10A | Tumor necrosis factor receptor superfamily member 10A | Immune response, Inflammatory response, Proteolysis |
| TNFRSF10C | Tumor necrosis factor receptor superfamily member 10C | Inflammatory response |
| TNFRSF11A | Tumor necrosis factor receptor superfamily member 11A | Immune response, Inflammatory response, MAPK cascade |
| TNFRSF13B | Tumor necrosis factor receptor superfamily member 13B | Immune response |
| TNFRSF14 | Tumor necrosis factor receptor superfamily member 14 | Cell adhesion, Inflammatory response, MAPK cascade |
| TNFSF13B | Tumor necrosis factor ligand superfamily member 13B | Other GO terms |
| t-PA | Tissue-type plasminogen activator | Coagulation, Proteolysis, Response to hypoxia, Wound healing |
| TR | Transferrin receptor protein 1 | Inflammatory response, Response to hypoxia |
| TRAIL-R2 | TNF-related apoptosis-inducing ligand receptor 2 | Immune response, Inflammatory response, Proteolysis |
| TR-AP | Tartrate-resistant acid phosphatase type 5 | Cell adhesion, Inflammatory response |
| uPA | Urokinase-type plasminogen activator | Angiogenesis, Blood vessel morphogenesis, Cell adhesion, Chemotaxis, Coagulation, Proteolysis, Response to hypoxia, Wound healing |
| U-PAR | Urokinase plasminogen activator surface receptor | Chemotaxis, Coagulation, Proteolysis, Wound healing |
| VEGFD | Vascular endothelial growth factor D | Angiogenesis, Blood vessel morphogenesis, Response to hypoxia |
| VSIG2 | V-set and immunoglobulin domain containing 2 | Other GO terms |
| vWF | von Willebrand factor | Cell adhesion, Coagulation, Platelet activation, Wound healing |
| XCL1 | Lymphotactin | Cell adhesion, Immune response, Inflammatory response, MAPK cascade |
