# Supplementary material for: Targeted proteomics of appendicular skeletal muscle mass and handgrip strength in black South Africans: a cross-sectional study
Source: Sci Rep. 2022 Jun 9;12:9512. doi: 10.1038/s41598-022-13548-9 (PMC9178538; doi:10.1038/s41598-022-13548-9)
Supplement: Supplementary file 3 — Supplementary Information 3. [file 41598_2022_13548_MOESM3_ESM.docx]

| **Biomarker** | **Normal Handgrip Strength Men** | | **Low Handgrip Strength Men** | | **P** |
| --- | --- | --- | --- | --- | --- |
|  | **N** | **Median (IQR)** | **N** | **Median (IQR)** |  |
| DLK-1 | 446 | 5.284 (4.808–5.694) | 18 | 5.566 (5.156–6.154) | 0.092 |
| GAL-9 | 434 | 7.898 (7.698–8.125) | 19 | 8.037 (7.911–8.284) | **0.007** |
| MEPE | 446 | 4.504 (4.232–4.753) | 18 | 4.471 (4.335–4.629) | 0.942 |
| MYOGLOBIN | 446 | 6.760 (6.335–7.152) | 18 | 6.963 (6.652–7.261) | 0.112 |
| SCF | 434 | 9.220 (8.865–9.458) | 19 | 9.167 (8.991–9.525) | 0.561 |

**Additional Table 10: All NPX comparison of the selected biomarkers between black South African men with normal and low handgrip strength.**

The Wilcoxon rank sum test was used to compare groups. **N:** Number of observations; **IQR:** Inter-quartile range; **P:** P value.
