# Supplementary material for: Targeted proteomics of appendicular skeletal muscle mass and handgrip strength in black South Africans: a cross-sectional study
Source: Sci Rep. 2022 Jun 9;12:9512. doi: 10.1038/s41598-022-13548-9 (PMC9178538; doi:10.1038/s41598-022-13548-9)
Supplement: Supplementary file 4 — Supplementary Information 4. [file 41598_2022_13548_MOESM4_ESM.docx]

**Additional Table 2: All tested associations between circulating protein biomarkers and appendicular skeletal muscle mass in the total sample comprising both men and women.**

| **Biomarker** | **Beta (95% CI)** | **P** | **FDR-Adjusted P** | **Sex Int P** |
| --- | --- | --- | --- | --- |
| ACE2 | -0.010 (-0.016, -0.003) | **0.003** | **0.019** | 0.848 |
| ADAM-TS13 | 0.001 (-0.033, 0.035) | 0.971 | 0.976 | 0.422 |
| ADM | -0.004 (-0.015, 0.008) | 0.539 | 0.760 | **7.29 e-05** |
| AGRP | -0.013 (-0.024, -0.002) | **0.016** | 0.066 | 0.197 |
| ALCAM | -0.009 (-0.023, 0.005) | 0.189 | 0.413 | **0.014** |
| AMBP | 0.005 (-0.020, 0.030) | 0.681 | 0.861 | 0.617 |
| ANGPT1 | 0.001 (-0.006, 0.008) | 0.838 | 0.936 | 0.399 |
| AP-N | -0.002 (-0.015, 0.011) | 0.715 | 0.874 | **0.008** |
| AXL | 0.011 (0.000, 0.023) | 0.056 | 0.170 | 0.069 |
| AZU1 | 0.002 (-0.004, 0.009) | 0.461 | 0.711 | 0.188 |
| BLM HYDROLASE | -0.005 (-0.012, 0.003) | 0.194 | 0.406 | 0.637 |
| BMP-6 | 0.001 (-0.005, 0.008) | 0.667 | 0.849 | 0.332 |
| BOC | -0.002 (-0.016, 0.012) | 0.790 | 0.915 | 0.708 |
| CA5A | -0.014 (-0.020, -0.009) | **1.33 e-07** | **3.46 e-06** | 0.344 |
| CASP-3 | -0.001 (-0.005, 0.003) | 0.757 | 0.912 | 0.236 |
| CCL15 | -0.002 (-0.010, 0.005) | 0.499 | 0.739 | 0.145 |
| CCL16 | -0.002 (-0.006, 0.002) | 0.392 | 0.636 | 0.916 |
| CCL17 | -0.001 (-0.006, 0.004) | 0.684 | 0.853 | 0.924 |
| CCL24 | 0.001 (-0.005, 0.008) | 0.666 | 0.854 | 0.958 |
| CCL3 | 0.000 (-0.003, 0.003) | 0.885 | 0.947 | 0.632 |
| CD163 | -0.002 (-0.010, 0.007) | 0.718 | 0.871 | **4.93 e-06** |
| CD4 | 0.000 (-0.012, 0.012) | 0.970 | 0.981 | 0.179 |
| CD40-L | 0.000 (-0.004, 0.004) | 0.937 | 0.958 | 0.423 |
| CD84 | -0.004 (-0.013, 0.005) | 0.355 | 0.609 | 0.337 |
| CD93 | 0.016 (0.004, 0.028) | **0.008** | **0.040** | 0.346 |
| CDH5 | 0.015 (0.005, 0.025) | **0.003** | **0.020** | 0.809 |
| CEACAM8 | -0.006 (-0.014, 0.002) | 0.117 | 0.294 | 0.115 |
| CHI3L1 | -0.010 (-0.013, -0.006) | **3.40 e-07** | **7.74 e-06** | **0.001** |
| CHIT1 | -0.004 (-0.008, -0.001) | **0.022** | 0.080 | 0.422 |
| CNTN1 | 0.015 (0.003, 0.026) | **0.016** | 0.065 | 0.822 |
| COL1A1 | -0.001 (-0.011, 0.009) | 0.786 | 0.917 | 0.057 |
| CPA1 | -0.015 (-0.021, -0.008) | **4.16 e-06** | **7.57 e-05** | 0.506 |
| CPB1 | -0.013 (-0.019, -0.007) | **4.36 e-05** | **4.96 e-04** | 0.349 |
| CSTB | 0.001 (-0.006, 0.007) | 0.873 | 0.951 | 0.341 |
| CTRC | -0.006 (-0.012, 0.000) | 0.057 | 0.170 | **3.68 e-05** |
| CTSD | -0.014 (-0.022, -0.006) | **0.001** | **0.008** | **0.003** |
| CTSL1 | -0.009 (-0.022, 0.005) | 0.219 | 0.442 | 0.155 |
| CTSZ | -0.002 (-0.014, 0.009) | 0.682 | 0.856 | **0.002** |
| CXCL1 | 0.001 (-0.005, 0.008) | 0.649 | 0.838 | 0.471 |
| CXCL16 | 0.015 (0.002, 0.027) | **0.021** | 0.076 | 0.101 |
| DCN | -0.005 (-0.023, 0.013) | 0.579 | 0.804 | **0.045** |
| DECR1 | -0.001 (-0.005, 0.004) | 0.817 | 0.929 | 0.859 |
| DKK-1 | -0.002 (-0.008, 0.004) | 0.538 | 0.765 | 0.456 |
| DLK-1 | 0.017 (0.009, 0.024) | **6.23 e-06** | **1.03 e-04** | 0.587 |
| EGFR | 0.022 (0.005, 0.039) | **0.011** | 0.050 | 0.616 |
| Ep-CAM | -0.008 (-0.014, -0.003) | **0.004** | **0.023** | 0.191 |
| EPHB4 | 0.003 (-0.010, 0.016) | 0.610 | 0.823 | **0.006** |
| FABP2 | 0.001 (-0.005, 0.008) | 0.647 | 0.841 | 0.200 |
| FABP4 | 0.015 (0.009, 0.022) | **6.41 e-06** | **9.72 e-05** | 0.364 |
| FAS | 0.005 (-0.003, 0.013) | 0.253 | 0.480 | 0.855 |
| FGF21 | -0.008 (-0.011, -0.005) | **3.87 e-08** | **1.41 e-06** | **0.003** |
| FGF-23 | 0.000 (-0.005, 0.004) | 0.929 | 0.961 | 0.790 |
| FS | -0.019 (-0.028, -0.010) | **4.44 e-05** | **4.75 e-04** | 0.238 |
| GAL-3 | -0.011 (-0.025, 0.002) | 0.104 | 0.283 | **0.006** |
| GAL-4 | -0.009 (-0.016, -0.002) | **0.012** | 0.055 | 0.322 |
| GAL-9 | -0.002 (-0.017, 0.013) | 0.764 | 0.914 | **0.023** |
| GDF-15 | -0.010 (-0.018, -0.003) | **0.007** | **0.036** | **0.006** |
| GDF-2 | -0.020 (-0.028, -0.012) | **4.05 e-07** | **8.19 e-06** | 0.456 |
| GH | -0.007 (-0.010, -0.005) | **7.78 e-08** | **2.36 e-06** | 0.112 |
| GIF | 0.001 (-0.003, 0.006) | 0.590 | 0.801 | 0.286 |
| GLO1 | -0.006 (-0.013, 0.001) | 0.109 | 0.291 | 0.793 |
| GP6 | -0.001 (-0.007, 0.004) | 0.641 | 0.840 | 0.311 |
| GRN | -0.005 (-0.018, 0.008) | 0.441 | 0.692 | **0.013** |
| GT | -0.001 (-0.007, 0.006) | 0.881 | 0.948 | 0.667 |
| HAOX1 | -0.006 (-0.009, -0.003) | **1.25 e-05** | **1.75 e-04** | 0.804 |
| HB-EGF | 0.002 (-0.002, 0.007) | 0.298 | 0.538 | 0.668 |
| HO-1 | -0.009 (-0.018, 0.000) | 0.051 | 0.158 | 0.289 |
| HOSCAR | -0.021 (-0.039, -0.004) | **0.018** | 0.071 | **0.025** |
| HSP 27 | 0.007 (-0.003, 0.018) | 0.174 | 0.395 | 0.584 |
| ICAM-2 | -0.005 (-0.017, 0.008) | 0.455 | 0.707 | **2.27 e-04** |
| IDUA | -0.006 (-0.015, 0.003) | 0.173 | 0.399 | 0.558 |
| IGFBP-1 | -0.017 (-0.021, -0.013) | **5.56 e-15** | **5.06 e-13** | 0.078 |
| IGFBP-2 | -0.021 (-0.027, -0.016) | **1.90 e-12** | **1.15 e-10** | **0.026** |
| IGFBP-7 | 0.001 (-0.010, 0.011) | 0.904 | 0.951 | **0.005** |
| IgG Fc receptor II-b | -0.005 (-0.013, 0.003) | 0.240 | 0.470 | 0.468 |
| IL16 | 0.010 (0.001, 0.019) | **0.033** | 0.109 | 0.683 |
| IL-17D | -0.045 (-0.187, 0.096) | 0.530 | 0.759 | 0.750 |
| IL17RA | -0.003 (-0.009, 0.003) | 0.330 | 0.578 | 0.889 |
| IL-18 | -0.003 (-0.010, 0.005) | 0.486 | 0.731 | 0.717 |
| IL-18BP | -0.007 (-0.019, 0.005) | 0.222 | 0.445 | **0.006** |
| IL-1RA | 0.005 (-0.003, 0.013) | 0.210 | 0.434 | 0.100 |
| IL1RL2 | 0.012 (0.004, 0.020) | **0.004** | **0.025** | 0.081 |
| IL-1RT1 | -0.009 (-0.021, 0.004) | 0.179 | 0.403 | **0.022** |
| IL-1RT2 | -0.008 (-0.017, 0.002) | 0.135 | 0.338 | **0.044** |
| IL-27 | -0.020 (-0.030, -0.010) | **1.08 e-04** | **0.001** | 0.093 |
| IL2-RA | -0.006 (-0.016, 0.003) | 0.184 | 0.409 | **0.009** |
| IL-4RA | -0.017 (-0.028, -0.006) | **0.003** | **0.019** | **0.001** |
| IL6 | -0.003 (-0.008, 0.002) | 0.215 | 0.440 | **1.70 e-05** |
| IL-6RA | 0.007 (-0.006, 0.019) | 0.313 | 0.559 | 0.960 |
| ITGB1BP2 | -0.001 (-0.004, 0.003) | 0.689 | 0.853 | 0.899 |
| ITGB2 | 0.003 (-0.006, 0.013) | 0.491 | 0.732 | 0.195 |
| JAM-A | -0.001 (-0.005, 0.003) | 0.503 | 0.738 | 0.144 |
| KIM1 | -0.004 (-0.010, 0.002) | 0.161 | 0.376 | 0.159 |
| KLK6 | -0.006 (-0.017, 0.005) | 0.271 | 0.498 | 0.193 |
| LDL receptor | 0.008 (0.001, 0.015) | **0.023** | 0.080 | 0.336 |
| LEP | 0.020 (0.015, 0.025) | **2.00 e-16** | **3.64 e-14** | **0.001** |
| LOX-1 | -0.007 (-0.016, 0.002) | 0.148 | 0.355 | 0.662 |
| LPL | 0.005 (-0.005, 0.015) | 0.362 | 0.616 | 0.869 |
| LTBR | 0.005 (-0.008, 0.018) | 0.428 | 0.684 | **0.013** |
| MARCO | 0.002 (-0.018, 0.022) | 0.828 | 0.930 | 0.127 |
| MCP-1 | -0.005 (-0.016, 0.006) | 0.368 | 0.614 | 0.151 |
| MEPE | 0.012 (0.002, 0.022) | **0.019** | 0.074 | **0.028** |
| MERTK | 0.002 (-0.006, 0.011) | 0.580 | 0.800 | 0.151 |
| MMP-12 | -0.012 (-0.020, -0.005) | **0.001** | **0.011** | 0.450 |
| MMP-2 | 0.006 (-0.006, 0.017) | 0.331 | 0.574 | 0.053 |
| MMP-3 | -0.006 (-0.014, 0.001) | 0.078 | 0.225 | **0.010** |
| MMP-7 | -0.011 (-0.025, 0.003) | 0.110 | 0.289 | 0.373 |
| MMP-9 | -0.001 (-0.008, 0.005) | 0.710 | 0.873 | 0.136 |
| MPO | 0.006 (-0.007, 0.018) | 0.369 | 0.611 | **0.009** |
| MYOGLOBIN | 0.019 (0.013, 0.026) | **2.49 e-08** | **1.13 e-06** | 0.115 |
| NEMO | 0.004 (-0.001, 0.008) | 0.143 | 0.347 | 0.467 |
| NOTCH3 | -0.005 (-0.015, 0.005) | 0.322 | 0.569 | **0.005** |
| NT-proBNP | -0.008 (-0.013, -0.004) | **4.88 e-04** | **0.004** | 0.507 |
| OPG | -0.015 (-0.026, -0.005) | **0.005** | **0.025** | **4.24 e-05** |
| OPN | -0.008 (-0.015, 0.000) | **0.038** | 0.120 | 0.924 |
| PAI | 0.002 (-0.003, 0.007) | 0.437 | 0.692 | 0.426 |
| PAPPA | -0.010 (-0.017, -0.002) | **0.010** | **0.046** | 0.241 |
| PAR-1 | 0.001 (-0.012, 0.013) | 0.897 | 0.949 | 0.847 |
| PARP-1 | -0.002 (-0.008, 0.003) | 0.427 | 0.688 | 0.360 |
| PCSK9 | -0.002 (-0.013, 0.010) | 0.783 | 0.920 | 0.330 |
| PDGF subunit A | 0.000 (-0.005, 0.005) | 0.931 | 0.957 | 0.724 |
| PDGF subunit B | 0.000 (-0.011, 0.011) | 0.983 | 0.983 | 0.794 |
| PD-L2 | -0.007 (-0.019, 0.005) | 0.252 | 0.483 | **0.037** |
| PECAM-1 | -0.002 (-0.007, 0.003) | 0.503 | 0.727 | 0.355 |
| PGF | 0.002 (-0.012, 0.015) | 0.824 | 0.932 | 0.258 |
| PGLYRP1 | -0.002 (-0.010, 0.006) | 0.620 | 0.830 | 0.081 |
| PI3 | 0.003 (-0.005, 0.010) | 0.481 | 0.729 | 0.050 |
| PIgR | -0.019 (-0.047, 0.009) | 0.190 | 0.412 | 0.288 |
| PLC | 0.032 (0.017, 0.047) | **2.24 e-05** | **2.91 e-04** | 0.714 |
| PON3 | -0.005 (-0.010, 0.001) | 0.078 | 0.222 | 0.219 |
| PRELP | -0.024 (-0.050, 0.001) | 0.059 | 0.172 | **0.007** |
| PRSS27 | -0.004 (-0.013, 0.005) | 0.365 | 0.615 | 0.368 |
| PRSS8 | -0.018 (-0.030, -0.006) | **0.003** | **0.019** | **0.012** |
| PRTN3 | -0.005 (-0.012, 0.003) | 0.246 | 0.477 | 0.590 |
| PSGL-1 | -0.002 (-0.010, 0.006) | 0.638 | 0.842 | 0.479 |
| PSP-D | -0.007 (-0.013, -0.001) | **0.020** | 0.074 | 0.165 |
| PTX3 | -0.016 (-0.024, -0.008) | **1.49 e-04** | **0.001** | 0.514 |
| RAGE | -0.011 (-0.022, -0.001) | **0.032** | 0.110 | **0.031** |
| RARRES2 | 0.018 (0.006, 0.031) | **0.005** | **0.026** | 0.979 |
| REN | -0.012 (-0.017, -0.006) | **4.25 e-05** | **5.16 e-04** | 0.478 |
| RETN | 0.003 (-0.004, 0.010) | 0.371 | 0.608 | 0.954 |
| SCF | 0.008 (-0.003, 0.018) | 0.139 | 0.343 | **0.001** |
| SCGB3A2 | -0.005 (-0.010, 0.000) | **0.036** | 0.115 | 0.205 |
| SELE | 0.000 (-0.009, 0.008) | 0.912 | 0.949 | **2.20 e-04** |
| SELP | -0.001 (-0.006, 0.005) | 0.791 | 0.905 | 0.374 |
| SERPINA12 | 0.003 (0.000, 0.006) | **0.032** | 0.109 | 0.528 |
| SHPS-1 | -0.006 (-0.016, 0.004) | 0.269 | 0.499 | 0.029 |
| SLAMF7 | -0.009 (-0.016, -0.002) | **0.010** | **0.046** | **3.54 e-04** |
| SOD2 | 0.006 (-0.019, 0.031) | 0.635 | 0.844 | 0.769 |
| SORT1 | -0.011 (-0.023, 0.002) | 0.086 | 0.237 | 0.810 |
| SPON2 | -0.022 (-0.049, 0.005) | 0.116 | 0.297 | **0.002** |
| SRC | -0.001 (-0.010, 0.008) | 0.891 | 0.949 | 0.230 |
| ST2 | -0.014 (-0.022, -0.007) | **8.63 e-05** | **0.001** | **0.002** |
| STK4 | 0.015 (0.004, 0.026) | **0.006** | **0.032** | 0.699 |
| TF | -0.013 (-0.024, -0.002) | 0.026 | 0.090 | 0.981 |
| TFF3 | 0.000 (-0.009, 0.008) | 0.910 | 0.952 | **0.007** |
| TFPI | -0.004 (-0.016, 0.008) | 0.503 | 0.733 | 0.778 |
| TGM2 | -0.005 (-0.015, 0.004) | 0.276 | 0.503 | 0.342 |
| THBS2 | -0.013 (-0.033, 0.007) | 0.194 | 0.411 | 0.550 |
| THPO | -0.002 (-0.010, 0.005) | 0.543 | 0.760 | 0.126 |
| TIE2 | 0.001 (-0.015, 0.018) | 0.862 | 0.950 | 0.813 |
| TIMP4 | -0.016 (-0.024, -0.008) | **1.45 e-04** | **0.001** | **1.36 e-04** |
| TLT-2 | 0.007 (-0.001, 0.015) | 0.082 | 0.229 | 0.560 |
| TM | 0.001 (-0.012, 0.014) | 0.858 | 0.952 | 0.589 |
| TNF-R1 | 0.014 (0.003, 0.025) | **0.015** | 0.065 | **0.004** |
| TNF-R2 | 0.001 (-0.009, 0.011) | 0.865 | 0.948 | **1.38 e-04** |
| TNFRSF10A | -0.021 (-0.034, -0.008) | **0.001** | **0.010** | **0.013** |
| TNFRSF10C | 0.001 (-0.009, 0.011) | 0.782 | 0.924 | 0.183 |
| TNFRSF11A | 0.004 (-0.007, 0.015) | 0.480 | 0.734 | **0.018** |
| TNFRSF13B | 0.006 (-0.003, 0.015) | 0.191 | 0.410 | 0.934 |
| TNFRSF14 | 0.000 (-0.007, 0.006) | 0.878 | 0.951 | 0.886 |
| TNFSF13B | 0.000 (-0.012, 0.011) | 0.967 | 0.984 | **0.001** |
| t-PA | -0.001 (-0.004, 0.003) | 0.791 | 0.911 | 0.159 |
| TR | -0.001 (-0.008, 0.006) | 0.766 | 0.911 | 0.492 |
| TRAIL-R2 | -0.016 (-0.029, -0.003) | **0.016** | 0.066 | **0.005** |
| TR-AP | -0.009 (-0.021, 0.002) | 0.112 | 0.290 | **0.030** |
| uPA | -0.008 (-0.018, 0.003) | 0.160 | 0.378 | 0.351 |
| U-PAR | -0.012 (-0.023, -0.001) | **0.033** | 0.108 | **0.010** |
| VEGFD | -0.023 (-0.037, -0.008) | **0.002** | **0.017** | 0.240 |
| VSIG2 | -0.005 (-0.013, 0.004) | 0.260 | 0.489 | 0.754 |
| vWF | -0.001 (-0.005, 0.003) | 0.589 | 0.806 | 0.926 |
| XCL1 | -0.005 (-0.014, 0.003) | 0.228 | 0.452 | 0.127 |

The linear regression models were adjusted for age, height, sex, smoking, alcohol, HFIAS total score, total physical activity, visceral adipose tissue, and HIV status. **Beta:** Unstandardized beta coefficient; **95 CI:** 95% confidence intervals; **P:** P value; **FDR-Adjusted P:** False Discovery Rate adjusted P value; **Sex Int P:** P value for sex interaction.
