# Supplementary material for: Targeted proteomics of appendicular skeletal muscle mass and handgrip strength in black South Africans: a cross-sectional study
Source: Sci Rep. 2022 Jun 9;12:9512. doi: 10.1038/s41598-022-13548-9 (PMC9178538; doi:10.1038/s41598-022-13548-9)
Supplement: Supplementary file 5 — Supplementary Information 5. [file 41598_2022_13548_MOESM5_ESM.docx]

**Additional Table 3: All tested associations between circulating protein biomarkers and appendicular skeletal muscle mass in the sex stratified linear regression models.**

| **Biomarker** | **Men** | | | **Women** | | |
| --- | --- | --- | --- | --- | --- | --- |
|  | **Beta (95% CI)** | **P** | **FDR-Adjusted P** | **Beta (95% CI)** | **P** | **FDR-Adjusted P** |
| ADM | -0.021 (-0.035, -0.007) | **0.003** | **0.006** | 0.026 (0.005, 0.047) | **0.014** | 0.075 |
| ALCAM | -0.026 (-0.044, -0.007) | **5.79 e-03** | **0.011** | 0.007 (-0.015, 0.030) | 0.513 | 0.785 |
| AP-N | -0.020 (-0.037, -0.002) | **0.028** | **0.037** | 0.013 (-0.007, 0.033) | 0.194 | 0.479 |
| CD163 | -0.018 (-0.028, -0.007) | **0.001** | **0.003** | 0.023 (0.009, 0.038) | **0.001** | **0.014** |
| CHI3L1 | -0.015 (-0.019, -0.010) | **2.08 e-10** | **2.70 e-09** | -0.002 (-0.008, 0.005) | 0.635 | 0.847 |
| CTRC | 0.005 (-0.003, 0.012) | 0.237 | 0.262 | -0.021 (-0.031, -0.011) | **2.16 e-05** | **0.001** |
| CTSD | -0.024 (-0.034, -0.013) | **9.42 e-06** | **5.44 e-05** | 0.000 (-0.014, 0.013) | 0.943 | 0.962 |
| CTSZ | -0.018 (-0.033, -0.003) | **0.016** | **0.024** | 0.016 (-0.002, 0.034) | 0.087 | 0.239 |
| DCN | -0.020 (-0.042, 0.002) | 0.070 | 0.085 | 0.016 (-0.016, 0.047) | 0.319 | 0.614 |
| EPHB4 | -0.014 (-0.031, 0.004) | 0.118 | 0.133 | 0.018 (-0.002, 0.038) | 0.074 | 0.213 |
| FGF21 | -0.011 (-0.014, -0.008) | **1.64 e-10** | **2.84 e-09** | 0.002 (-0.008, 0.004) | 0.504 | 0.794 |
| GAL-3 | -0.025 (-0.041, -0.009) | **0.003** | **0.006** | 0.011 (-0.013, 0.034) | 0.359 | 0.667 |
| GAL-9 | -0.016 (-0.034, 0.003) | 0.091 | 0.105 | 0.018 (-0.007, 0.043) | 0.155 | 0.403 |
| GDF-15 | -0.018 (-0.027, -0.009) | **6.94 e-05** | **3.01 e-04** | 0.004 (-0.010, 0.017) | 0.598 | 0.840 |
| GRN | -0.019 (-0.035, -0.002) | **0.028** | **0.037** | 0.011 (-0.009, 0.030) | 0.279 | 0.558 |
| HOSCAR | -0.038 (-0.059, -0.017) | **4.82 e-04** | **0.002** | 0.004 (-0.027, 0.035) | 0.815 | 0.865 |
| ICAM-2 | -0.025 (-0.041, -0.009) | **0.003** | **0.006** | 0.019 (-0.001, 0.038) | 0.060 | 0.195 |
| IGFBP-2 | -0.029 (-0.036, -0.021) | **2.53 e-13** | **6.58 e-12** | -0.014 (-0.023, -0.004) | **0.006** | **0.043** |
| IGFBP-7 | -0.012 (-0.025, 0.001) | 0.075 | 0.089 | 0.017 (-0.001, 0.035) | 0.067 | 0.204 |
| IL-18BP | -0.022 (-0.037, -0.007) | **0.005** | **0.010** | 0.008 (-0.011, 0.027) | 0.409 | 0.685 |
| IL-1RT1 | -0.023 (-0.040, -0.006) | **0.010** | **0.016** | 0.002 (-0.017, 0.021) | 0.814 | 0.881 |
| IL-1RT2 | -0.016 (-0.029, -0.004) | **0.012** | **0.019** | 0.003 (-0.013, 0.019) | 0.702 | 0.811 |
| IL2-RA | -0.016 (-0.028, -0.004) | **0.007** | **0.012** | 0.006 (-0.009, 0.021) | 0.407 | 0.705 |
| IL-4RA | -0.032 (-0.046, -0.018) | **5.59 e-06** | **3.63 e-05** | 0.006 (-0.014, 0.026) | 0.544 | 0.809 |
| IL6 | -0.009 (-0.015, -0.004) | **0.001** | **0.004** | 0.017 (0.006, 0.028) | **0.003** | **0.027** |
| LEP | 0.021 (0.015, 0.026) | **2.24 e-13** | **1.16 e-11** | 0.031 (0.021, 0.042) | **7.18 e-09** | **3.73 e-07** |
| LTBR | -0.009 (-0.026, 0.007) | 0.274 | 0.296 | 0.020 (0.000, 0.039) | 0.051 | 0.176 |
| MEPE | 0.022 (0.009, 0.036) | **0.001** | **0.004** | -0.003 (-0.019, 0.013) | 0.697 | 0.823 |
| MMP3 | -0.016 (-0.025, -0.006) | **0.001** | **0.003** | 0.002 (-0.008, 0.013) | 0.667 | 0.867 |
| MPO | -0.008 (-0.024, 0.008) | 0.333 | 0.346 | 0.022 (0.003, 0.041) | **0.022** | 0.103 |
| NOTCH3 | -0.017 (-0.029, -0.005) | **0.007** | **0.012** | 0.011 (-0.006, 0.027) | 0.204 | 0.482 |
| OPG | -0.036 (-0.049, -0.022) | **4.49 e-07** | **3.34 e-06** | 0.007 (-0.009, 0.023) | 0.363 | 0.651 |
| PD-L2 | -0.018 (-0.034, -0.003) | **0.018** | **0.027** | 0.005 (-0.014, 0.025) | 0.598 | 0.863 |
| PRELP | -0.053 (-0.084, -0.023) | **0.001** | **0.002** | 0.018 (-0.026, 0.062) | 0.422 | 0.686 |
| PRSS8 | -0.031 (-0.046, -0.016) | **7.28 e-05** | **2.91 e-04** | -0.003 (-0.022, 0.015) | 0.731 | 0.827 |
| RAGE | -0.004 (-0.017, 0.009) | 0.524 | 0.534 | -0.031 (-0.049, -0.012) | **0.001** | **0.014** |
| SCF | 0.018 (0.006, 0.030) | **0.003** | **0.006** | -0.021 (-0.041, -0.001) | **0.041** | 0.165 |
| SELE | -0.013 (-0.024, -0.002) | **0.017** | **0.025** | 0.017 (0.004, 0.029) | **0.010** | 0.059 |
| SHPS-1 | -0.015 (-0.028, -0.002) | **0.021** | **0.029** | 0.004 (-0.012, 0.020) | 0.616 | 0.844 |
| SLAMF7 | -0.019 (-0.027, -0.011) | **1.01 e-05** | **5.25 e-05** | 0.007 (-0.005, 0.019) | 0.251 | 0.521 |
| SPON2 | -0.052 (-0.084, -0.020) | **0.002** | **0.004** | 0.031 (-0.018, 0.080) | 0.212 | 0.478 |
| ST2 | -0.023 (-0.032, -0.014) | **2.69 e-07** | **2.33 e-06** | -0.002 (-0.015, 0.011) | 0.756 | 0.836 |
| TFF3 | -0.016 (-0.030, -0.001) | **0.031** | **0.040** | 0.007 (-0.004, 0.018) | 0.218 | 0.471 |
| TIMP4 | -0.029 (-0.039, -0.019) | **3.63 e-08** | **3.78 e-07** | 0.003 (-0.011, 0.017) | 0.676 | 0.857 |
| TNF-R1 | -0.002 (-0.017, 0.013) | 0.816 | 0.816 | 0.031 (0.013, 0.048) | **0.001** | **0.014** |
| TNF-R2 | -0.015 (-0.027, -0.002) | **0.019** | **0.026** | 0.023 (0.006, 0.039) | **0.007** | **0.048** |
| TNFRSF10A | -0.032 (-0.047, -0.017) | **3.13 e-05** | **1.48 e-04** | 0.001 (-0.023, 0.025) | 0.954 | 0.954 |
| TNFRSF11A | -0.007 (-0.021, 0.006) | 0.299 | 0.317 | 0.020 (0.001, 0.039) | **0.042** | 0.156 |
| TNFSF13B | -0.015 (-0.028, -0.001) | **0.040** | **0.049** | 0.021 (0.002, 0.040) | **0.031** | 0.133 |
| TRAILR2 | -0.030 (-0.045, -0.015) | **1.47 e-04** | **0.001** | 0.005 (-0.018, 0.027) | 0.689 | 0.834 |
| TR-AP | -0.020 (-0.035, -0.006) | **0.007** | **0.012** | 0.002 (-0.018, 0.021) | 0.861 | 0.896 |
| U-PAR | -0.022 (-0.036, -0.009) | **0.001** | **0.004** | 0.004 (-0.014, 0.022) | 0.676 | 0.837 |

The linear regression models were adjusted for age, height, sex, smoking, alcohol, HFIAS total score, total physical activity, visceral adipose tissue, and HIV status. Menopause status was included as an additional confounder in women only. **Beta:** Unstandardized beta coefficient; **95:** 95% confidence intervals; **P:** P value; **FDR-Adjusted P:** False Discovery Rate adjusted P value.
