# Supplementary material for: Targeted proteomics of appendicular skeletal muscle mass and handgrip strength in black South Africans: a cross-sectional study
Source: Sci Rep. 2022 Jun 9;12:9512. doi: 10.1038/s41598-022-13548-9 (PMC9178538; doi:10.1038/s41598-022-13548-9)
Supplement: Supplementary file 6 — Supplementary Information 6. [file 41598_2022_13548_MOESM6_ESM.docx]

**Additional Table 4: All NPX comparison of the selected biomarkers between black South Africans with normal and low appendicular skeletal muscle mass in the total sample comprising both men and women.**

| **Biomarker** | **Normal ASM** | | **Low ASM** | | **P** |
| --- | --- | --- | --- | --- | --- |
|  | **N** | **Median (IQR)** | **N** | **Median (IQR)** |  |
| ACE2 | 799 | 3.851 (3.408–4.363) | 69 | 3.778 (3.370–4.210) | 0.416 |
| CA5A | 758 | 2.591 (2.160–3.243) | 68 | 2.558 (2.178–2.888) | 0.520 |
| CD93 | 820 | 10.637 (10.380–10.864) | 67 | 10.530 (10.306–10.722) | **0.009** |
| CDH5 | 820 | 3.306 (3.050–3.635) | 67 | 3.240 (2.913–3.539) | 0.086 |
| CHI3L1 | 820 | 6.423 (5.711–7.366) | 67 | 6.411 (5.732–7.090) | 0.557 |
| CPA1 | 820 | 5.196 (4.721–5.723) | 68 | 5.287 (4.910–5.615) | 0.569 |
| CPB1 | 820 | 5.599 (5.101–6.143) | 68 | 5.795 (5.295–6.053) | 0.135 |
| CTSD | 820 | 3.634 (3.337–4.030) | 67 | 3.629 (3.386–4.090) | 0.701 |
| DLK-1 | 820 | 5.299 (4.875–5.739) | 67 | 5.218 (4.715–5.784) | 0.243 |
| Ep-CAM | 820 | 5.253 (4.771–5.906) | 67 | 5.335 (4.779–5.968) | 0.767 |
| FABP4 | 820 | 5.077 (4.368–5.732) | 67 | 5.500 (5.077–5.998) | **2.68 e-05** |
| FGF21 | 799 | 7.630 (6.731–8.624) | 69 | 7.787 (6.946–8.848) | 0.463 |
| FS | 799 | 11.305 (10.957–11.592) | 69 | 11.347 (11.113–11.681) | 0.307 |
| GDF-15 | 820 | 4.989 (4.614–5.456) | 67 | 5.071 (4.720–5.408) | 0.413 |
| GDF-2 | 799 | 9.155 (8.703–9.540) | 69 | 9.031 (8.761–9.369) | 0.436 |
| GH | 799 | 7.839 (6.402–9.284) | 69 | 7.936 (6.196–8.791) | 0.525 |
| HAOX1 | 799 | 5.475 (4.479–6.904) | 69 | 5.327 (4.746–6.681) | 0.965 |
| IGFBP-1 | 820 | 6.152 (5.362–6.976) | 67 | 5.896 (5.027–6.553) | **0.032** |
| IGFBP-2 | 820 | 8.068 (7.457–8.695) | 67 | 7.890 (7.309–8.376) | **0.045** |
| IL1RL2 | 799 | 4.045 (3.570–4.389) | 69 | 4.046 (3.847–4.328) | 0.400 |
| IL27 | 799 | 5.985 (5.696–6.256) | 69 | 5.896 (5.609–6.277) | 0.420 |
| IL-4RA | 799 | 2.293 (2.124–2.537) | 69 | 2.255 (2.025–2.427) | 0.064 |
| LEP | 790 | 6.394 (4.936–7.276) | 69 | 7.262 (6.547–7.708) | **6.92 e-07** |
| MMP-12 | 799 | 7.850 (7.478–8.291) | 69 | 7.830 (7.367–8.194) | 0.505 |
| MYOGLOBIN | 820 | 6.583 (6.216–7.040) | 67 | 6.433 (6.032–6.818) | 0.061 |
| NT-proBNP | 758 | 3.064 (2.432–3.846) | 61 | 2.817 (2.077–3.472) | **0.034** |
| OPG | 820 | 3.669 (3.398–3.980) | 67 | 3.652 (3.486–3.828) | 0.875 |
| PAPPA | 799 | 3.384 (2.998–3.801) | 69 | 3.233 (2.969–3.350) | 0.377 |
| PLC | 820 | 7.807 (7.602–8.002) | 67 | 7.845 (7.659–8.011) | 0.705 |
| PRSS8 | 799 | 8.695 (8.377–9.005) | 69 | 8.697 (8.426–8.980) | 0.911 |
| PTX3 | 799 | 3.321 (2.964–3.639) | 69 | 3.426 (3.093–3.722) | 0.106 |
| RARRES2 | 820 | 11.770 (11.540–11.980) | 67 | 11.852 (11.669–12.051) | **0.036** |
| REN | 799 | 6.040 (5.489–6.618) | 69 | 6.092 (5.447–6.694) | 0.433 |
| SLAMF7 | 799 | 4.036 (3.653–4.556) | 69 | 4.000 (3.585–4.330) | 0.175 |
| ST2 | 820 | 6.242 (5.869–6.671) | 67 | 6.275 (5.845–6.685) | 0.770 |
| STK4 | 799 | 5.771 (5.535–5.908) | 69 | 5.732 (5.435–5.881) | 0.106 |
| TIMP4 | 820 | 4.593 (4.208–4.994) | 67 | 4.693 (4.240–5.074) | 0.443 |
| TNFRSF10A | 799 | 3.366 (3.139–3.576) | 69 | 3.408 (3.154–3.564) | 0.876 |
| VEGFD | 799 | 7.915 (7.706–8.134) | 69 | 7.790 (7.655–8.103) | 0.078 |

The Wilcoxon rank sum test was used to compare groups. **ASM:** Appendicular Skeletal Muscle Mass; **N:** Number of observations; **IQR:** Inter-quartile range; **P:** P value.
