# Supplementary material for: Targeted proteomics of appendicular skeletal muscle mass and handgrip strength in black South Africans: a cross-sectional study
Source: Sci Rep. 2022 Jun 9;12:9512. doi: 10.1038/s41598-022-13548-9 (PMC9178538; doi:10.1038/s41598-022-13548-9)
Supplement: Supplementary file 7 — Supplementary Information 7. [file 41598_2022_13548_MOESM7_ESM.docx]

**Additional Table 5: All NPX comparison of the selected biomarkers between black South African men with normal and low appendicular skeletal muscle mass.**

| **Biomarker** | **Normal ASM Men** | | **Low ASM Men** | | **P** |
| --- | --- | --- | --- | --- | --- |
|  | **N** | **Median (IQR)** | **N** | **Median (IQR)** |  |
| ADM | 407 | 8.231 (7.984–8.493) | 30 | 8.531 (8.128–8.643) | **0.009** |
| ALCAM | 418 | 7.239 (7.037–7.432) | 29 | 7.197 (6.987–7.418) | 0.734 |
| APN | 418 | 5.500 (5.298–5.740) | 29 | 5.444 (5.248–5.576) | 0.184 |
| CD163 | 418 | 7.714 (7.380–8.076) | 29 | 7.707 (7.470–8.242) | 0.572 |
| CHI3L1 | 418 | 6.393 (5.652–7.455) | 29 | 6.087 (5.328–7.049) | 0.246 |
| CTSD | 418 | 3.641 (3.356–4.063) | 29 | 3.629 (3.437–3.970) | 0.864 |
| CTSZ | 418 | 4.293 (4.007–4.541) | 29 | 4.401 (3.974–4.626) | 0.860 |
| FGF21 | 407 | 7.764 (6.864–8.787) | 30 | 8.204 (7.370–9.440) | 0.069 |
| GAL-3 | 418 | 4.855 (4.627–5.076) | 29 | 5.010 (4.813–5.250) | **0.010** |
| GDF-15 | 418 | 5.111 (4.709–5.560) | 29 | 5.289 (4.998–5.697) | 0.121 |
| GRN | 418 | 5.250 (5.042–5.481) | 29 | 5.178 (5.034–5.415) | 0.554 |
| HOSCAR | 407 | 10.740 (10.557–10.905) | 30 | 10.770 (10.510–10.920) | 0.950 |
| ICAM-2 | 418 | 5.493 (5.255–5.718) | 29 | 5.441 (5.326–5.791) | 0.824 |
| IGFBP-2 | 418 | 8.242 (7.556–8.876) | 29 | 7.897 (7.509–8.389) | 0.109 |
| IL-18BP | 418 | 5.960 (5.751–6.227) | 29 | 5.998 (5.846–6.252) | 0.649 |
| IL-1RT1 | 418 | 5.684 (5.479–5.905) | 29 | 5.764 (5.491–5.828) | 0.854 |
| IL-1RT2 | 418 | 5.600 (5.323–5.906) | 30 | 5.603 (5.462–5.934) | 0.943 |
| IL-2RA | 418 | 3.933 (3.628–4.274) | 29 | 3.912 (3.774–4.317) | 0.574 |
| IL-4RA | 407 | 2.314 (2.129–2.555) | 30 | 2.257 (2.044–2.513) | 0.470 |
| IL6 | 407 | 3.697 (3.249–4.208) | 30 | 4.099 (3.390–5.029) | **0.042** |
| LEP | 398 | 5.027 (3.645–6.012) | 30 | 6.236 (5.261–6.854) | **2.13 e-05** |
| MEPE | 418 | 4.500 (4.230–4.752) | 29 | 4.530 (4.347–4.638) | 0.840 |
| MMP3 | 418 | 6.997 (6.599–7.362) | 29 | 6.919 (6.543–7.462) | 0.953 |
| NOTCH3 | 418 | 5.329 (5.013–5.602) | 29 | 5.186 (4.800–5.566) | 0.239 |
| OPG | 418 | 3.600 (3.315–3.873) | 29 | 3.609 (3.480–3.784) | 0.959 |
| PDL2 | 407 | 3.586 (3.345–3.855) | 30 | 3.567 (3.278–3.847) | 0.830 |
| PRELP | 407 | 8.384 (8.271–8.491) | 30 | 8.389 (8.225–8.537) | 0.968 |
| PRSS8 | 407 | 8.847 (8.540–9.068) | 30 | 8.899 (8.664–9.216) | 0.274 |
| SCF | 407 | 9.230 (8.900–9.466) | 30 | 9.014 (8.684–9.296) | **0.027** |
| SELE | 418 | 10.514 (10.178–10.907) | 29 | 10.744 (10.566–11.118) | **0.014** |
| SHPS-1 | 418 | 3.664 (3.354–3.955) | 30 | 3.526 (3.185–3.848) | 0.362 |
| SLAMF7 | 407 | 4.018 (3.595–4.583) | 30 | 4.061 (3.700–4.433) | 0.948 |
| SPON2 | 407 | 8.258 (8.149–8.361) | 30 | 8.230 (8.147–8.358) | 0.953 |
| ST2 | 418 | 6.443 (6.045–6.851) | 29 | 6.481 (6.172–6.911) | 0.567 |
| TFF3 | 418 | 6.250 (5.987–6.517) | 29 | 6.284 (6.084–6.503) | 0.554 |
| TIMP4 | 418 | 4.366 (4.028–4.761) | 29 | 4.477 (4.111–4.902) | 0.560 |
| TNF-R2 | 418 | 5.234 (4.955–5.532) | 29 | 5.345 (5.107–5.641) | 0.372 |
| TNFRSF10A | 407 | 3.345 (3.098–3.609) | 30 | 3.409 (3.035–3.651) | 0.692 |
| TNFSF13B | 418 | 6.844 (6.549–7.101) | 29 | 6.877 (6.747–7.011) | 0.615 |
| TRAIL-R2 | 407 | 6.194 (5.944–6.404) | 30 | 6.319 (6.026–6.536) | 0.074 |
| TR-AP | 418 | 5.104 (4.833–5.360) | 29 | 5.160 (4.875–5.258) | 0.893 |
| U-PAR | 418 | 5.157 (4.837–5.433) | 29 | 5.193 (4.988–5.584) | 0.491 |

The Wilcoxon rank sum test was used to compare groups. **ASM:** Appendicular Skeletal Muscle Mass; **N:** Number of observations; **IQR:** Inter-quartile range; **P:** P value.
