# Supplementary material for: Targeted proteomics of appendicular skeletal muscle mass and handgrip strength in black South Africans: a cross-sectional study
Source: Sci Rep. 2022 Jun 9;12:9512. doi: 10.1038/s41598-022-13548-9 (PMC9178538; doi:10.1038/s41598-022-13548-9)
Supplement: Supplementary file 8 — Supplementary Information 8. [file 41598_2022_13548_MOESM8_ESM.docx]

**Additional Table 6: All NPX comparison of the selected biomarkers between women with normal and low appendicular skeletal muscle mass.**

| **Biomarker** | **Normal ASM Women** | | **Low ASM Women** | | **P** |
| --- | --- | --- | --- | --- | --- |
|  | **N** | **Median (IQR)** | **N** | **Median (IQR)** |  |
| CD163 | 402 | 7.934 (7.553–8.277) | 38 | 7.897 (7.611–8.216) | 0.901 |
| CTRC | 392 | 10.537 (9.963–11.002) | 39 | 10.674 (10.177–11.116) | 0.273 |
| IGFBP-2 | 402 | 7.947 (7.404–8.474) | 38 | 7.853 (7.245–8.299) | 0.301 |
| IL6 | 392 | 3.858 (3.439–4.361) | 39 | 4.168 (3.733–4.588) | **0.015** |
| LEP | 392 | 7.252 (6.739–7.660) | 39 | 7.665 (7.343–7.939) | **4.77 e-05** |
| RAGE | 392 | 13.020 (12.700–13.320) | 39 | 12.820 (12.570–13.160) | **0.016** |
| TNF-R1 | 402 | 6.309 (6.042–6.577) | 38 | 6.282 (6.054–6.394) | 0.197 |
| TNF-R2 | 402 | 5.277 (5.009–5.572) | 38 | 5.200 (4.865–5.409) | 0.069 |

The Wilcoxon rank sum test was used to compare groups. **ASM:** Appendicular Skeletal Muscle Mass; **N:** Number of observations; **IQR:** Inter-quartile range; **P:** P value.
