# Supplementary material for: Targeted proteomics of appendicular skeletal muscle mass and handgrip strength in black South Africans: a cross-sectional study
Source: Sci Rep. 2022 Jun 9;12:9512. doi: 10.1038/s41598-022-13548-9 (PMC9178538; doi:10.1038/s41598-022-13548-9)
Supplement: Supplementary file 9 — Supplementary Information 9. [file 41598_2022_13548_MOESM9_ESM.docx]

**Additional Table 7: All tested associations between circulating protein biomarkers and handgrip strength in the total sample comprising both men and women.**

| **Biomarker** | **Beta (95% CI)** | **P** | **FDR-Adjusted P** | **Sex Int P** |
| --- | --- | --- | --- | --- |
| ACE2 | -0.001 (-0.072, 0.071) | 0.983 | 0.994 | 0.434 |
| ADAM-TS13 | -0.055 (-0.426, 0.316) | 0.770 | 0.898 | 0.771 |
| ADM | -0.105 (-0.232, 0.022) | 0.105 | 0.376 | 0.749 |
| AGRP | -0.110 (-0.228, 0.007) | 0.066 | 0.315 | 0.543 |
| ALCAM | -0.030 (-0.184, 0.123) | 0.698 | 0.858 | 0.713 |
| AMBP | -0.189 (-0.463, 0.086) | 0.178 | 0.433 | 0.200 |
| ANGPT1 | -0.075 (-0.150, 0.000) | **0.049** | 0.260 | 0.561 |
| AP-N | -0.029 (-0.170, 0.112) | 0.687 | 0.851 | 0.795 |
| AXL | 0.023 (-0.103, 0.148) | 0.724 | 0.872 | 0.668 |
| AZU1 | 0.035 (-0.035, 0.111) | 0.323 | 0.566 | 0.105 |
| BLM HYDROLASE | -0.066 (-0.147, 0.016) | 0.113 | 0.367 | 0.517 |
| BMP-6 | -0.024 (-0.092, 0.043) | 0.482 | 0.731 | 0.418 |
| BOC | 0.010 (-0.147, 0.168) | 0.898 | 0.967 | 0.838 |
| CA5A | -0.029 (-0.088, 0.031) | 0.345 | 0.586 | 0.520 |
| CASP-3 | -0.030 (-0.075, 0.015) | 0.186 | 0.440 | 0.679 |
| CCL15 | -0.021 (-0.101, 0.058) | 0.595 | 0.785 | 0.985 |
| CCL16 | -0.003 (-0.048, 0.043) | 0.906 | 0.947 | 0.140 |
| CCL17 | -0.061 (-0.113, -0.010) | **0.020** | 0.176 | 0.092 |
| CCL24 | 0.021 (-0.050, 0.092) | 0.563 | 0.788 | 0.950 |
| CCL3 | -0.014 (-0.048, 0.019) | 0.404 | 0.656 | 0.061 |
| CD163 | 0.012 (-0.081, 0.104) | 0.806 | 0.906 | 0.478 |
| CD4 | -0.197 (-0.327, -0.067) | **0.003** | 0.060 | 0.932 |
| CD40-L | -0.035 (-0.079, 0.009) | 0.122 | 0.390 | 0.523 |
| CD84 | -0.044 (-0.139, 0.051) | 0.366 | 0.605 | 0.763 |
| CD93 | -0.017 (-0.148, 0.114) | 0.800 | 0.910 | 0.185 |
| CDH5 | 0.022 (-0.086, 0.131) | 0.686 | 0.855 | 0.440 |
| CEACAM8 | -0.114 (-0.198, -0.030) | **0.008** | 0.093 | 0.858 |
| CHI3L1 | -0.043 (-0.083, -0.002) | **0.041** | 0.239 | 0.194 |
| CHIT1 | -0.015 (-0.054, 0.024) | 0.448 | 0.697 | 0.179 |
| CNTN1 | 0.115 (-0.014, 0.243) | 0.081 | 0.367 | 0.052 |
| COL1A1 | -0.009 (-0.119, 0.102) | 0.879 | 0.958 | 0.264 |
| CPA1 | -0.002 (-0.071, 0.067) | 0.948 | 0.980 | 0.736 |
| CPB1 | 0.000 (-0.068, 0.068) | 0.990 | 0.996 | 0.563 |
| CSTB | -0.039 (-0.115, 0.037) | 0.316 | 0.570 | 0.967 |
| CTRC | 0.012 (-0.053, 0.078) | 0.716 | 0.868 | 0.115 |
| CTSD | -0.059 (-0.150, 0.033) | 0.209 | 0.431 | 0.505 |
| CTSL1 | -0.069 (-0.218, 0.080) | 0.365 | 0.610 | 0.282 |
| CTSZ | -0.039 (-0.162, 0.084) | 0.534 | 0.765 | 0.284 |
| CXCL1 | -0.033 (-0.100, 0.034) | 0.330 | 0.572 | 0.238 |
| CXCL16 | -0.045 (-0.182, 0.092) | 0.515 | 0.756 | 0.554 |
| DCN | -0.013 (-0.209, 0.184) | 0.899 | 0.962 | 0.611 |
| DECR1 | -0.042 (-0.091, 0.007) | 0.090 | 0.372 | 0.529 |
| DKK-1 | -0.053 (-0.121, 0.016) | 0.134 | 0.394 | 0.664 |
| DLK-1 | 0.056 (-0.022, 0.135) | 0.160 | 0.410 | **0.001** |
| EGFR | 0.222 (0.037, 0.406) | **0.019** | 0.169 | 0.140 |
| Ep-CAM | -0.051 (-0.110, 0.009) | 0.099 | 0.366 | 0.584 |
| EPHB4 | -0.039 (-0.180, 0.103) | 0.592 | 0.792 | 0.794 |
| FABP2 | 0.034 (-0.032, 0.101) | 0.311 | 0.567 | 0.212 |
| FABP4 | 0.041 (-0.032, 0.113) | 0.270 | 0.523 | 0.259 |
| FAS | 0.011 (-0.077, 0.100) | 0.802 | 0.906 | 0.834 |
| FGF21 | -0.015 (-0.048, 0.018) | 0.377 | 0.617 | 0.382 |
| FGF-23 | -0.021 (-0.072, 0.030) | 0.413 | 0.665 | 0.146 |
| FS | -0.133 (-0.232, -0.034) | **0.008** | 0.095 | 0.384 |
| GAL-3 | -0.109 (-0.256, 0.038) | 0.146 | 0.396 | 0.281 |
| GAL-4 | -0.031 (-0.109, 0.048) | 0.446 | 0.700 | 0.672 |
| GAL-9 | -0.189 (-0.352, -0.026) | **0.023** | 0.193 | **0.040** |
| GDF-15 | -0.128 (-0.209, -0.047) | **0.002** | **0.045** | 0.642 |
| GDF-2 | -0.141 (-0.226, -0.056) | **0.001** | **0.029** | 0.636 |
| GH | -0.021 (-0.050, 0.009) | 0.175 | 0.430 | 0.534 |
| GIF | 0.015 (-0.035, 0.065) | 0.557 | 0.791 | 0.086 |
| GLO1 | -0.058 (-0.137, 0.021) | 0.148 | 0.395 | 0.771 |
| GP6 | -0.034 (-0.095, 0.026) | 0.263 | 0.514 | 0.455 |
| GRN | -0.015 (-0.154, 0.124) | 0.832 | 0.924 | 0.127 |
| GT | -0.010 (-0.086, 0.065) | 0.790 | 0.910 | 0.811 |
| HAOX1 | -0.003 (-0.034, 0.028) | 0.835 | 0.921 | 0.498 |
| HB-EGF | -0.009 (-0.060, 0.042) | 0.728 | 0.872 | 0.420 |
| HO-1 | -0.028 (-0.127, 0.072) | 0.582 | 0.791 | 0.363 |
| HOSCAR | -0.156 (-0.348, 0.036) | 0.112 | 0.369 | 0.283 |
| HSP 27 | 0.116 (0.000, 0.231) | **0.049** | 0.256 | 0.268 |
| ICAM-2 | -0.122 (-0.259, 0.015) | 0.082 | 0.364 | 0.668 |
| IDUA | -0.029 (-0.128, 0.070) | 0.565 | 0.779 | 0.845 |
| IGFBP-1 | -0.091 (-0.138, -0.044) | **1.35 e-04** | **0.008** | 0.399 |
| IGFBP-2 | -0.161 (-0.226, -0.096) | **1.32 e-06** | **2.40 e-04** | 0.768 |
| IGFBP-7 | 0.022 (-0.094, 0.138) | 0.714 | 0.872 | 0.611 |
| IgG Fc receptor II-b | -0.057 (-0.148, 0.034) | 0.218 | 0.446 | 0.979 |
| IL16 | 0.034 (-0.065, 0.134) | 0.496 | 0.746 | 0.899 |
| IL-17D | -0.045 (-0.187, 0.096) | 0.530 | 0.765 | 0.746 |
| IL17RA | -0.040 (-0.106, 0.026) | 0.232 | 0.464 | 0.567 |
| IL-18 | 0.013 (-0.067, 0.093) | 0.750 | 0.887 | 0.857 |
| IL-18BP | -0.116 (-0.246, 0.015) | 0.083 | 0.358 | 0.760 |
| IL-1RA | 0.000 (-0.084, 0.084) | 0.997 | 0.997 | 0.189 |
| IL1RL2 | 0.021 (-0.069, 0.111) | 0.644 | 0.831 | 0.560 |
| IL-1RT1 | -0.107 (-0.246, 0.031) | 0.129 | 0.391 | 0.559 |
| IL-1RT2 | -0.033 (-0.015, 0.202) | 0.092 | 0.363 | 0.627 |
| IL-27 | -0.206 (-0.317, -0.096) | **2.57 e-04** | **0.009** | 0.810 |
| IL2-RA | -0.084 (-0.185, 0.016) | 0.101 | 0.367 | 0.270 |
| IL-4RA | -0.186 (-0.309, -0.063) | **0.003** | 0.052 | 0.312 |
| IL6 | -0.059 (-0.114, -0.004) | **0.035** | 0.230 | 0.688 |
| IL-6RA | -0.009 (-0.151, 0.134) | 0.905 | 0.952 | 0.924 |
| ITGB1BP2 | -0.039 (-0.076, -0.002) | **0.041** | 0.246 | 0.316 |
| ITGB2 | 0.101 (-0.005, 0.206) | 0.061 | 0.301 | 0.644 |
| JAM-A | -0.022 (-0.064, 0.019) | 0.296 | 0.561 | 0.699 |
| KIM1 | -0.088 (-0.149, -0.026) | **0.005** | 0.074 | 0.931 |
| KLK6 | 0.039 (-0.083, 0.161) | 0.528 | 0.769 | 0.053 |
| LDL receptor | 0.080 (0.006, 0.153) | **0.034** | 0.230 | 0.354 |
| LEP | 0.059 (0.006, 0.111) | **0.028** | 0.223 | 0.835 |
| LOX-1 | -0.107 (-0.211, -0.002) | **0.045** | 0.249 | 0.069 |
| LPL | -0.003 (-0.114, 0.108) | 0.959 | 0.981 | 0.572 |
| LTBR | -0.144 (-0.284, -0.005) | **0.043** | 0.245 | 0.508 |
| MARCO | -0.051 (-0.267, 0.165) | 0.644 | 0.826 | 0.851 |
| MCP-1 | -0.078 (-0.198, 0.041) | 0.199 | 0.437 | 0.632 |
| MEPE | 0.171 (0.058, 0.284) | **0.003** | 0.055 | **0.007** |
| MERTK | 0.022 (-0.072, 0.115) | 0.645 | 0.821 | 0.678 |
| MMP-12 | -0.083 (-0.166, 0.001) | 0.053 | 0.268 | 0.933 |
| MMP-2 | -0.031 (-0.155, 0.093) | 0.623 | 0.810 | 0.306 |
| MMP-3 | -0.051 (-0.129, 0.027) | 0.200 | 0.434 | 0.718 |
| MMP-7 | -0.285 (-0.437, -0.133) | **2.45 e-04** | **0.011** | 0.695 |
| MMP-9 | -0.047 (-0.118, 0.025) | 0.203 | 0.430 | 0.642 |
| MPO | 0.009 (-0.122, 0.141) | 0.888 | 0.962 | 0.563 |
| MYOGLOBIN | 0.060 (-0.016, 0.137) | 0.124 | 0.389 | **0.022** |
| NEMO | -0.028 (-0.081, 0.025) | 0.297 | 0.557 | 0.482 |
| NOTCH3 | -0.099 (-0.206, 0.007) | 0.068 | 0.316 | 0.974 |
| NT-proBNP | -0.098 (-0.147, -0.049) | **1.06 e-04** | **0.010** | 0.810 |
| OPG | -0.058 (-0.173, 0.057) | 0.321 | 0.573 | 0.708 |
| OPN | -0.110 (-0.189, -0.031) | **0.007** | 0.087 | 0.687 |
| PAI | -0.039 (-0.097, 0.019) | 0.188 | 0.427 | 0.305 |
| PAPPA | -0.070 (-0.150, 0.010) | 0.087 | 0.368 | 0.221 |
| PAR-1 | -0.036 (-0.170, 0.098) | 0.599 | 0.784 | 0.867 |
| PARP-1 | 0.002 (-0.060, 0.064) | 0.961 | 0.977 | 0.967 |
| PCSK9 | 0.029 (-0.100, 0.159) | 0.657 | 0.830 | 0.477 |
| PDGF subunit A | -0.042 (-0.093, 0.009) | 0.109 | 0.373 | 0.347 |
| PDGF subunit B | -0.088 (-0.205, 0.029) | 0.140 | 0.386 | 0.358 |
| PD-L2 | -0.096 (-0.228, 0.035) | 0.151 | 0.397 | 0.590 |
| PECAM-1 | -0.036 (-0.090, 0.018) | 0.188 | 0.432 | 0.467 |
| PGF | -0.101 (-0.252, 0.050) | 0.188 | 0.423 | 0.889 |
| PGLYRP1 | -0.033 (-0.124, 0.057) | 0.468 | 0.716 | 0.896 |
| PI3 | -0.056 (-0.139, 0.028) | 0.191 | 0.423 | 0.735 |
| PIgR | -0.106 (-0.418, 0.206) | 0.505 | 0.754 | 0.685 |
| PLC | 0.005 (-0.160, 0.171) | 0.949 | 0.976 | 0.677 |
| PON3 | 0.027 (-0.031, 0.085) | 0.363 | 0.612 | 0.181 |
| PRELP | -0.177 (-0.453, 0.099) | 0.207 | 0.434 | 0.202 |
| PRSS27 | -0.027 (-0.122, 0.069) | 0.584 | 0.787 | 0.939 |
| PRSS8 | -0.091 (-0.221, 0.038) | 0.167 | 0.423 | 0.829 |
| PRTN3 | -0.062 (-0.148, 0.024) | 0.156 | 0.406 | 0.758 |
| PSGL-1 | -0.012 (-0.097, 0.073) | 0.785 | 0.910 | 0.423 |
| PSP-D | 0.033 (-0.030, 0.096) | 0.298 | 0.554 | 0.653 |
| PTX3 | -0.103 (-0.195, -0.011) | **0.029** | 0.218 | 0.254 |
| RAGE | 0.006 (-0.109, 0.121) | 0.914 | 0.950 | 0.685 |
| RARRES2 | -0.070 (-0.209, 0.069) | 0.322 | 0.568 | 0.775 |
| REN | -0.010 (-0.071, 0.052) | 0.756 | 0.888 | 0.730 |
| RETN | -0.066 (-0.142, 0.011) | 0.091 | 0.368 | 0.837 |
| SCF | 0.077 (-0.037, 0.190) | 0.184 | 0.441 | **0.008** |
| SCGB3A2 | -0.004 (-0.058, 0.051) | 0.899 | 0.952 | 0.911 |
| SELE | -0.053 (-0.142, 0.036) | 0.239 | 0.474 | 0.979 |
| SELP | -0.037 (-0.094, 0.020) | 0.201 | 0.430 | 0.700 |
| SERPINA12 | 0.009 (-0.022, 0.039) | 0.564 | 0.784 | 0.950 |
| SHPS-1 | -0.066 (-0.174, 0.042) | 0.231 | 0.466 | 0.441 |
| SLAMF7 | -0.051 (-0.127, 0.025) | 0.186 | 0.435 | 0.202 |
| SOD2 | 0.150 (-0.123, 0.423) | 0.282 | 0.540 | 0.163 |
| SORT1 | -0.170 (-0.304, -0.036) | **0.013** | 0.124 | 0.314 |
| SPON2 | -0.391 (-0.687, -0.095) | **0.010** | 0.103 | 0.807 |
| SRC | 0.048 (-0.050, 0.146) | 0.334 | 0.574 | 0.847 |
| ST2 | -0.119 (-0.198, -0.040) | **0.003** | **0.048** | 0.168 |
| STK4 | -0.039 (-0.157, 0.078) | 0.511 | 0.755 | 0.307 |
| TF | -0.085 (-0.206, 0.036) | 0.169 | 0.421 | 0.260 |
| TFF3 | -0.101 (-0.194, -0.008) | **0.033** | 0.228 | 0.214 |
| TFPI | 0.009 (-0.124, 0.141) | 0.899 | 0.957 | 0.255 |
| TGM2 | 0.042 (-0.062, 0.147) | 0.428 | 0.684 | 0.525 |
| THBS2 | -0.029 (-0.248, 0.190) | 0.793 | 0.908 | 0.748 |
| THPO | -0.068 (-0.149, 0.012) | 0.097 | 0.369 | 0.443 |
| TIE2 | 0.030 (-0.148, 0.207) | 0.744 | 0.885 | 0.819 |
| TIMP4 | -0.168 (-0.258, -0.077) | **2.97 e-04** | **0.009** | 0.911 |
| TLT-2 | 0.009 (-0.080, 0.098) | 0.844 | 0.925 | 0.418 |
| TM | -0.053 (-0.192, 0.085) | 0.448 | 0.691 | 0.953 |
| TNF-R1 | -0.094 (-0.218, 0.030) | 0.138 | 0.399 | 0.569 |
| TNF-R2 | -0.091 (-0.198, 0.016) | 0.095 | 0.369 | 0.368 |
| TNFRSF10A | -0.109 (-0.251, 0.032) | 0.128 | 0.395 | 0.260 |
| TNFRSF10C | -0.083 (-0.192, 0.027) | 0.138 | 0.393 | 0.940 |
| TNFRSF11A | -0.133 (-0.253, -0.012) | **0.031** | 0.223 | 0.341 |
| TNFRSF13B | 0.023 (-0.078, 0.123) | 0.660 | 0.829 | 0.255 |
| TNFRSF14 | -0.052 (-0.121, 0.017) | 0.140 | 0.391 | 0.662 |
| TNFSF13B | -0.101 (-0.224, 0.022) | 0.107 | 0.373 | 0.103 |
| t-PA | -0.011 (-0.053, 0.030) | 0.594 | 0.788 | 0.237 |
| TR | -0.021 (-0.093, 0.050) | 0.562 | 0.793 | **0.044** |
| TRAIL-R2 | -0.181 (-0.321, -0.041) | **0.011** | 0.114 | 0.627 |
| TR-AP | -0.036 (-0.164, 0.091) | 0.576 | 0.788 | 0.316 |
| uPA | -0.046 (-0.163, 0.071) | 0.445 | 0.705 | 0.672 |
| U-PAR | -0.124 (-0.242, -0.005) | **0.040** | 0.254 | 0.075 |
| VEGFD | -0.124 (-0.284, 0.036) | 0.130 | 0.388 | 0.889 |
| VSIG2 | -0.076 (-0.168, 0.017) | 0.110 | 0.372 | 0.719 |
| vWF | -0.021 (-0.062, 0.020) | 0.307 | 0.565 | 0.335 |
| XCL1 | -0.011 (-0.105, 0.083) | 0.820 | 0.916 | 0.937 |

The linear regression models were adjusted for age, height, sex, smoking, alcohol, HFIAS total score, total physical activity, visceral adipose tissue, and HIV status. **Beta:** Unstandardized beta coefficient; **95CI:** 95% confidence intervals; **P:** P value; **FDR-Adjusted P:** False Discovery Rate adjusted P value; **Sex Int P:** P value for sex interaction.
