# Supplementary material for: Targeted proteomics of appendicular skeletal muscle mass and handgrip strength in black South Africans: a cross-sectional study
Source: Sci Rep. 2022 Jun 9;12:9512. doi: 10.1038/s41598-022-13548-9 (PMC9178538; doi:10.1038/s41598-022-13548-9)
Supplement: Supplementary file 11 — Supplementary Information 11. [file 41598_2022_13548_MOESM11_ESM.docx]

| **Biomarker** | **Normal Handgrip Strength** | | **Low Handgrip Strength** | | **P** |
| --- | --- | --- | --- | --- | --- |
|  | **N** | **Median (IQR)** | **N** | **Median (IQR)** |  |
| GDF-15 | 867 | 4.998 (4.617–5.449) | 44 | 4.944 (4.475–5.466) | 0.428 |
| GDF-2 | 846 | 9.142 (8.693–9.538) | 46 | 8.820 (8.618–9.155) | **0.002** |
| IGFBP-1 | 867 | 6.130 (5.290–6.969) | 44 | 5.568 (4.892–6.113) | **4.64 e-04** |
| IGFBP-2 | 867 | 8.043 (7.436–8.686) | 44 | 7.559 (7.022–7.992) | **0.002** |
| IL27 | 846 | 5.982 (5.683–6.263) | 46 | 5.982 (5.644–6.280) | 0.883 |
| MMP-7 | 846 | 10.300 (10.090–10.490) | 46 | 10.371 (10.124–10.527) | 0.377 |
| NT-proBNP | 798 | 3.053 (2.403–3.807) | 38 | 3.116 (2.466–4.093) | 0.257 |
| TIMP4 | 867 | 4.583 (4.197–4.962) | 44 | 4.941 (4.368–5.187) | **0.017** |

**Additional Table 9: All NPX comparison of the selected biomarkers between black South Africans with normal and low handgrip strength in the total sample comprising both men and women.**

The Wilcoxon rank sum test was used to compare groups. **N:** Number of observations; **IQR:** Inter-quartile range; **P:** P value.
